# Three-Month Administration of PB125 Modifies Histopathology, Redox Homeostasis, and Mobility in the Hartley Guinea Pig Model of Primary Osteoarthritis

**DOI:** 10.3390/antiox15020212

**Published:** 2026-02-05

**Authors:** Kendra M. Andrie, Robert V. Musci, Maureen A. Walsh, Sydney Bork, Zachary J. Valenti, Joseph Sanford, Margaret Campbell, Leila F. Afzali, Maryam F. Afzali, Karyn L. Hamilton, Kelly S. Santangelo

**Affiliations:** 1 Department of Microbiology, Immunology, and Pathology, Colorado State University, Fort Collins, CO 80523, USA; kendra.andrie@colostate.edu (K.M.A.); joseph.sanford@colostate.edu (J.S.); margaret.campbell@colostate.edu (M.C.); mary.afzali@colostate.edu (M.F.A.); 2 Department of Health and Exercise Science, Colorado State University, Fort Collins, CO 80523, USA; robert.musci@colostate.edu (R.V.M.); maureen.walsh@colostate.edu (M.A.W.); zachary.valenti@colostate.edu (Z.J.V.); karyn.hamilton@colostate.edu (K.L.H.); 3 Department of Statistics, Colorado State University, Fort Collins, CO 80523, USA; leila.afzali@colostate.edu; 4 Center for Healthy Aging, Colorado State University, Fort Collins, CO 80523, USA

**Keywords:** Nrf2/ARE signaling, Dunkin Hartley guinea pig, osteoarthritis, cartilage, infrapatellar fat pad/synovial complex, PB125

## Abstract

The pathogenesis of primary osteoarthritis (OA) is complex and multifactorial. Nuclear factor erythroid 2-related factor-2 (Nrf2) is a transcription factor that regulates hundreds of genes involved with cytoprotection. The role of Nrf2 in OA remains undefined. We utilized the Hartley guinea pig model of primary OA to investigate the role of a purported Nrf2 activator, PB125, in delaying the onset of knee OA. We hypothesized that three months of daily PB125 supplementation would modify structural, molecular, and in vivo functional outcomes characteristic of disease. Fifty-six 2-month-old animals (equal sexes) were treated orally with PB125 or vehicle control for 3 months; animals were sacrificed at 5 months, which represents mild OA and early disease. Outcome measures included knee histopathology, mRNA expression, immunohistochemistry, and in vivo mobility. Notably, PB125 treatment had differing effects in males and females. Female PB125-treated animals had significantly decreased distal femur OA scores, accompanied by differential gene and protein expression patterns in articular cartilage for markers related to redox homeostasis; decreases in one compulsory mobility metric were also seen. In contrast, males demonstrated a statistical difference in voluntary mobility patterns. In summary, PB125 may modify the molecular mechanisms involved in the initiation of early OA in a potential sex-dependent fashion.

## 1. Introduction

Osteoarthritis (OA) is a degenerative joint disease and is the most common cause of disability in older adults, affecting up to 30 million people and 30–50% of some demographics [1,2,3]. Primary OA is considered relatively spontaneous, with no clear underlying traumatic injury or recognized hypersensitivity reaction driving local degeneration within the joint cavity. (This is in contrast to “secondary” OA, which is most often associated with an overt inciting cause, such as direct injury or trauma to the joint.) The pathogenesis of primary OA is thus complex, multifactorial, and largely undetermined [4]. A major limitation in managing OA is the lack of therapeutics that can delay or prevent the onset or slow the progression of disease. Given this, interventions typically focus on pain modification and are often of limited efficacy, which can result in immobility and depression [5].

Hyaline cartilage is unique, as it is avascular, alymphatic, aneural, and paucicellular and thus has limited renewal capacity once damaged. There is a growing body of evidence linking an age-related imbalance with increased reactive oxygen species, chondrocyte death, and articular cartilage degeneration [6]. Nrf2 is a transcription factor that, when activated by stress (oxidative, inflammatory, or energetic), binds the antioxidant response element (ARE) and serves as a master regulator of hundreds of cytoprotective genes, including anti-inflammatory agents, phase I xenobiotics, and phase II antioxidant enzymes [6,7,8,9]. Functional alterations in Nrf2 signaling are implicated in a vast array of pathologies, including carcinogenesis and chemoresistance, as well as cardiovascular and neurodegenerative diseases [10,11,12,13,14,15,16]. In vivo studies have demonstrated that Nrf2 activation is protective to cartilage and other joint tissues in various murine models of post-traumatic OA; Nrf2-knockout mice also demonstrate more severe cartilage degeneration and accelerated overall disease progression in secondary (or post-traumatic) OA models [17,18,19,20]. However, there is a knowledge gap regarding the role of Nrf2 in the development of the progressive chondro-degenerative phenotype seen in primary OA and, specifically, in the Hartley guinea pig model of joint disease.

We have previously published work supporting the potential benefits of a purported phytochemical Nrf2 agonist, PB125, in skeletal muscle and long bones [21,22]. PB125 is a patented, registered, commercially available dietary supplement that contains three active ingredients—rosemary extract (6.7% carnosol, R), ashwagandha extract (1% withaferin A), and sophora japonica (98% luteolin, L)—which have previously been shown to both activate and stabilize Nrf2 [23].

Given our promising findings in other musculoskeletal tissues in vivo, we aimed to determine the influence of PB125 on a diathrodial joint organ. As chondrocytes have limited capacity to respond to oxidative damage that accumulates with age, we took the approach of preemptive treatment with PB125 to influence structural, molecular, and clinical manifestations of early-stage disease. We theorized that dysregulation in Nrf2/ARE signaling within articular cartilage and peri-articular musculoskeletal tissue may serve as a driver for persistent low-grade inflammatory signaling; this, in turn, may contribute to the early onset of knee OA characteristic of this model. More specifically, we hypothesized that animals treated with PB125 before the onset of knee OA would have decreased knee joint OA scores. Secondary outcomes of interest included differential gene expression profiles within articular cartilage and the infrapatellar fat pad/synovium complex (IFP/SC) and modified clinical parameters, including differential mobility patterns and voluntary activity levels, during gait analysis and enclosure monitoring, respectively.

It is noted that the presence of sexual dimorphism in OA has been increasingly highlighted, yet many pivotal animal studies have only been conducted in males [24]. Furthermore, when treatments are employed in preclinical models, males and females tend to have differing responses to the same treatment [25]. Thus, given an increasing need to have preclinical interventions include both males and females, our approach was powered to detect and describe any sex-specific treatment differences.

## 2. Materials and Methods

### 2.1. Experimental Animals and Design

Procedures were approved by the Colorado State University (CSU) Institutional Animal Care and Use Committee (Protocol #19-9129A) and were performed in accordance with the NIH Guide for the Care and Use of Laboratory Animals. Hartley guinea pigs were procured from Charles River Laboratories (Wilmington, MA, USA). Male (*n* = 28) and female (*n* = 28) 1.5-month-old (mo) animals were acquired to carry out a prospective placebo-controlled, PB125 treatment study. As a comparison group to identify age and sex-specific differences in OA histology, as well as changes in aggrecan and Nrf2 expression, 28 additional 15 mo old animals (*n* = 14/sex) were purchased. Animals were maintained at CSU Laboratory Animal Resources housing facilities and were monitored daily by veterinarians. The guinea pigs were singly housed in solid-bottom cages with red huts to provide an enriched environment and *ad libitum* access to water and a regular chow diet. Hay cubes were withheld to ensure that the main source of phytochemicals was limited to the oral Nrf2 supplement.

After a 2-week acclimation period, animals included in the prospective study were randomly assigned to receive treatment with PB125^®^ (Pathways Bioscience, Denver, CO, USA) or the delivery vehicle control (ORA-sweet^®^ Sugar Free Syrup Vehicle, Paddock Laboratories, Minneapolis, MN, USA). PB125-treated guinea pigs received 8 mg/kg of compound suspended in 1 mg/mL of ORA-sweet SF vehicle, while controls received a volume-based vehicle equivalent [21]. This same dose and route of administration have been utilized in this animal model previously [21,22]; of note, a single lot/batch of PB125 was used throughout the study. Animals began treatment at 2 months of age; guinea pigs were dosed daily in the morning for 3 months and sacrificed at 5 months of age, when mild OA changes representing early-stage disease were expected in control animals [26,27]. Details pertinent to animal body weight, activity levels, and diet consumption have been reported [21,22].

### 2.2. Euthanasia and Tissue Acquisition

One control male guinea pig was euthanized early due to an injury that was not associated with the study intervention; as this occurred close to the end of the study, the data was included in all outcomes. The remaining animals were anesthetized with a mixture of isoflurane and oxygen and transferred to a carbon dioxide chamber for euthanasia. Complete necropsy was performed by a veterinary pathologist to rule out co-morbidities. The right hindlimb was disarticulated at the coxofemoral joint. Upon arthrotomy, the articular surfaces (patellar, patella-femoral groove, femoral condylar, and tibial plateau) of these knees were scraped and pooled for storage in RNALater. The IFP/SC was removed and stored in Allprotect Tissue Reagent. All tissues were stored at −80 °C until further processing. The left hindlimb was removed, left intact, stored in situ in 10% neutral buffered formalin for 48 h, and transferred to PBS.

### 2.3. Histopathology

The left hindlimbs were removed from PBS and decalcified over 6 weeks in Ca^2+^EDTA. The limbs were trimmed in the medial sagittal plane, embedded in cassettes, and processed; 5µM paraffin sections were mounted onto charged slides. The slides were stained with toluidine blue for Osteoarthritis Research Society International (OARSI) grading, as per Kraus et al. [28]. Knee joints were scored in random order by a single board-certified veterinary pathologist (KMA), who was blinded to treatment group. All animals were included in this outcome.

### 2.4. NanoString mRNA Gene Expression

A custom NanoString nCounter 100-code gene panel for targeted mRNA analysis was utilized, consisting of Nrf2 and Nrf2-induced genes, as well as key mediators of inflammation and OA pathogenesis. Total mRNA, from pooled articular cartilage or the IFP of the right knee joint, was extracted using Qiagen RNeasy and Rneasy Lipid Mini Kits, respectively, in accordance with the manufacturer’s instructions. Samples were analyzed in collaboration with Nanostring Technologies at the University of Arizona Genetics Core. Per initial RNA quantification (Invitrogen Qubit 2.0 Fluorometer and RNA High Sensitivity Assay Kit, Thermo Fisher Scientific, Waltham, MA, USA) and Fragment Analyzer quality control subsets (Fragment Analyzer Automated CE System and High Sensitivity RNA Assay Kit, Agilent Technologies, Santa Clara, CA, USA), the optimal amount of total RNA (800.00 ng) was hybridized with the custom code set in an overnight incubation set to 65 °C, followed by processing on the NanoString nCounter FLEX Analysis system. The results were reported as absolute transcript counts normalized to positive controls and one housekeeping gene, eukaryotic translation elongation factor 1 alpha 1. Any potential sample input variance was corrected by use of the housekeeping gene and application of a sample-specific correction factor in all target probes. Data analysis was conducted using nSolver software (NanoString Technologies, Bothell, WA, USA). A complete gene list and normalized mRNA counts from the articular cartilage and IFP/SC are provided in Supplemental Tables S1 and S2, respectively. For this Supplemental Data, analyses were performed using two-way ANOVA (factors signifying sex and treatment), with adjusted *p* values determined via more conservative Bonferroni corrections for multiple comparisons. In some cases, animals were excluded from final statistical analyses due to not passing quality control expectations associated with either initial RNA extraction or faulty sample input variance.

### 2.5. Immunohistochemistry (IHC)

To characterize selected gene expression outcomes at the protein level, IHC was implemented using antibodies against NAD(P)H/quinone oxidoreducatase 1 (NQO1) (Abcam, Cambridge, UK; ab34173, rabbit polyclonal, dilution of 1:400) and Nrf2 (ThermoFisher Scientific, Waltham, MA, USA; PA-38312, rabbit polyclonal, dilution of 1:100). Heat-induced epitope retrieval was performed on a Leica Bond-RX Automated Stainer using BOND Epitope Retrieval Solution 1 (citrate buffer) for 420 min at 55 °C. Blocking was performed using 3% peroxide followed by 2.5% donkey serum. Labeling was performed on an automated staining platform. DAB was used as the chromogen, and immunoreactions were visualized using commercial detection systems. Slides were counterstained with hematoxylin. The sequential steps of the immunostaining procedure were also performed on negative controls using secondary antibodies only. Given that both adipocytes and synoviocytes were included in the evaluation of immunoreactivity within the IFP/SC, a semi-quantitative grading scheme was utilized: tissues were graded on a scale of 0–8 by assessing the percentage of positive cells (0–5: 0% = 0; <5% = 1; 6–24% = 2; 25–49% = 3; 50–74% = 4; and >75% = 5) combined with the intensity of immunolabeling (0–3: absent = 0; slight = 1; moderate = 2; and marked = 3) [29]. As only chondrocytes were evaluated, articular cartilage photomicrographs were quantitatively analyzed by measuring the mean DAB immuno-positive surface area and percentage of positive cells via thresholding in VisioPharm software version 2025.08.01 (Westminster, CO, USA). Some animals were excluded from these analyses due to tissue loss during the processing steps, even after repeated attempts at the assay.

### 2.6. Compulsory Treadmill-Based Gait Analysis

The animals were acclimated over a 2-week period before the onset of the study to a treadmill-based gait analysis system (DigiGait^TM^, Mouse Specifics, Framingham, MA, USA). The guinea pigs were run on a flat treadmill at 55 cm/s, and videos were analyzed for 22 gait parameters; analyses focused on mean propel time. See Supplemental Table S3 for a full list of output variables and descriptive statistics. Some animals were excluded from this outcome, as they were not amenable to walking on the treadmill.

### 2.7. Overhead Enclosure Monitoring

Before the onset of the study, the animals were acclimated over a 2-week period to an open circular field behavior monitoring system (ANY-maze^TM^, Wood Dale, IL, USA) to assess voluntary physical mobility. Activity was recorded monthly during the same time period of each day (8 A.M.–12 P.M.) for 10 consecutive minutes throughout the study. Analyses were focused on the following parameters: total distance traveled (m), average speed (m/s), time mobile (s), % time mobile, time in huts (s), and % time in huts. See Supplemental Table S4 for a full list of output variables and descriptive statistics. All animals were included in this outcome.

### 2.8. Statistical Analyses

Stride length from computer-assisted gait analysis was identified as the primary outcome to determine the appropriate sample size. The rationale for this selection was that this parameter was anticipated to require the highest sample size to achieve statistical power, thereby ensuring adequate numbers for all other measures [30]. A previous study demonstrated that changes in stride length with anti-inflammatory treatment had an average effect size of 2.6 cm [31]. For the current study, we set an effect size of 1 cm for the power calculation, a power of 0.80, and significance (chance of Type I error) at *p* = 0.05 (Russ Lenth power calculator). Based on these values, n = 13 animals per treatment group was identified; n = 14/group was selected to account for unexpected loss.

**Figure 1:**
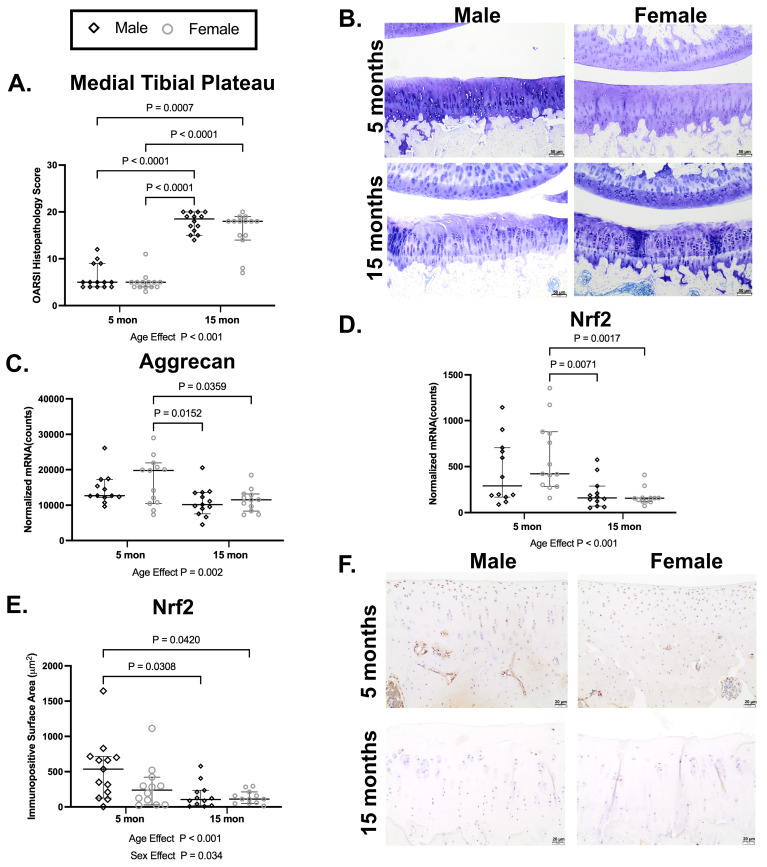
Older 15mo Hartley guinea pigs have worsened OA accompanied with lower aggrecan and Nrf2 expression. (**A**) Fifteen-month-old male and female Hartley guinea pigs have higher medial tibial plateau OARSI histopathology scores for OA than 5 mo animals (median difference for males: 13.5 units; median difference for females: 13 units). (**B**) Representative medial tibial plateau toluidine blue photomicrographs from 5-month (top row) and 15-month (bottom row) male (left column) and female (right column) guinea pigs. Femoral condyles and tibial plateaus are on the top and bottom of each image, respectively. Toluidine blue, 10× objective, bar = 50 μm. (**C**) There was an overall age effect of aggrecan mRNA expression, which was statistically lower in female guinea pigs at 15 mo than at 5 mo (mean difference: −5378 counts). (**D**) Nrf2 mRNA expression was also lower at 15 mo than at 5 mo; this effect was predominantly driven by females (median difference for females: −264 counts). (**E**) Supporting the mRNA findings, there was an age and sex effect for the Nrf2 mean immunopositive surface area of the medial tibial plateau; interestingly, this statistical effect was prominent in males (median difference for males: 431.3 mm^2^). (**F**) Representative Nrf2 IHC photomicrographs of the medial tibial plateau from 5mo (top row) and 15mo (bottom row) male (left column) and female (right column) Hartley guinea pigs. 20× objective, bar = 20 μm.

**Figure 2:**
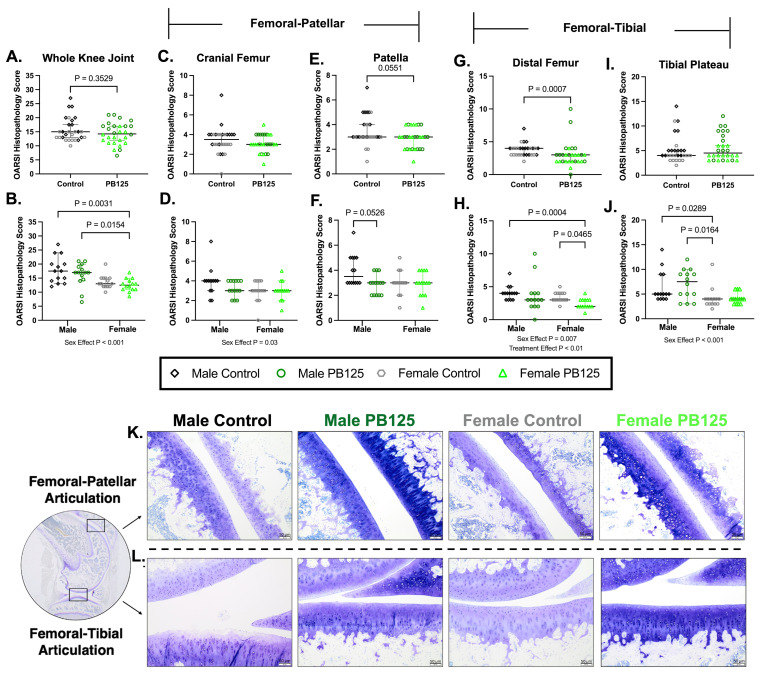
Distal femur OA scores were statistically improved with PB125 treatment in females but not in males. (**A**,**B**) When separated or combined by sex, a main effect of sex was present in whole knee joint scores, but no treatment-related differences were found. (**C**,**D**) No significant differences related to treatment were detected in the cranial femur regardless of whether data was combined or separated by sex. (**E**,**F**) A near significant effect of PB125 was detected in the patellar surface when sexes were combined (median difference: 0.50); this appeared to be associated with a near significant difference between treated and control males (median difference: 0.50). (**G**) Data with sexes combined for distal femur scoring showed a statistical difference between control and treated animals (mean difference: −1.00). (**H**) Distal femur OA scores were significantly lower with PB125 treatment in females (median difference: −1.00) but not in males. (**I**,**J**) No significant differences related to treatment were detected in the tibial plateau regardless of whether data was combined or separated by sex. There was a significant difference between control male and female animals in this structure (median difference: 1.00). (**K**,**L**) (Left) Subgross photomicrograph of the guinea pig whole knee joint trimmed in the sagittal plane; black boxes depict graded regions of interest, including the femoral-patellar and femoral-tibial articular surfaces. Representative photomicrographs of the femoral-patellar articulation (top row, (**K**)) and the femoral-tibial articulation (bottom row, (**L**)). Toluidine blue, 10× objective, bar = 50 μm.

Normality assessment was performed via the Shapiro–Wilk test on all outcome measures. For all tests, significance was set to *p* ≤ 0.05; *p* values were reported in the text for *p* < 0.06. Parametric and nonparametric data combined for sex were analyzed using either unpaired *t*-tests or Mann–Whitney statistics, respectively. Parametric data separated by treatment and sex, including transcript changes and compulsory mobility, were expressed as mean ± 95% confidence intervals (CIs). These outcomes were analyzed using two-way ANOVA (factors signifying sex and treatment), followed by Tukey–Kramer multiple comparison *post hoc* tests; no specific correction for multiple comparisons were applied in these scenarios. OARSI histopathology scores (Figure 1A and Figure 2A–J), semi-quantitative IHC scores, and other nonparametric data were expressed as median ± 95% CIs. These datasets were analyzed using a two-factor nonparametric factorial model (ART ANOVA), which allowed the testing of main effects (e.g., sex, treatment, or age) and their interaction while remaining robust to non-normal distributions and unequal variance.

All analyses, save one, were performed using Prism 10.4.1 (GraphPad, San Diego, CA, USA); ART ANOVA was accomplished with R version 4.5.2 (R Foundation for Statistical Computing, Vienna, Austria). Figures were constructed with Prism; mean/median effect sizes (differences) are indicated in the figure legends.

## 3. Results and Discussion

### 3.1. Aging Effects in Non-Treated Hartley Guinea Pigs: Older 15 Mo Hartley Guinea Pigs Have More Severe OA Accompanied with Lower Aggrecan and Nrf2 Expression (Figure 1)

Hartley guinea pigs of both sexes have higher medial tibial plateau OARSI scores for OA at 15 mo than at 5 mo, which is consistent with prior work (main effect of age *p* < 0.001; Figure 1A,B) [32]. In support of impaired cartilage homeostasis at older ages, there is a main effect of age (*p* = 0.002) on aggrecan mRNA expression, which was statistically lower (*p* = 0.0359) in female guinea pigs at 15 mo than at 5 mo (Figure 1C). There was also an age effect (*p* < 0.001) on Nrf2 mRNA expression; this, again, was driven by a difference between older and younger females (*p* = 0.0017; Figure 1D). Supporting the mRNA findings, there were main effects of both age (*p* < 0.001) and sex (*p* = 0.034) on the reduction in the Nrf2 mean immunopositive surface area of the medial tibial plateau in 15 mo animals (Figure 1E); in this case, a statistical difference was present between male guinea pigs (*p* = 0.0308). Cumulatively, we demonstrated that transcript and/or protein expression of the oxidative/stress-sensing transcription factor Nrf2 declines in vivo from 5 to 15 months of age in articular cartilage, which provides justification for attempting to modulate this factor.

### 3.2. OARSI OA Scores: OA Scores Were Statistically Improved with PB125 Treatment in Females but Not in Males (Figure 2)

At the end of the 3 mo treatment study, 5 mo control females had lower median OA scores for the tibial plateau than 5 mo control males.(*p* = 0.0289, Figure 2J), which is similar to trends previously reported in this strain [32].

With sexes combined, statistically significant differences related to treatment were detected in the distal femur (Figure 2G). When separated by sex, this finding was driven by a statistical difference between control and PB125-treated females (*p* = 0.0465; Figure 2H). No significant treatment effects were noted with whole knee joint (Figure 2A,B), cranial femur (Figure 2C,D), patella (*p* = 0.0551 and *p* = 0.0526; Figure 2E,F, respectively), or tibial plateau (Figure 2I,J) OA scores. Sagittal subgross and representative photomicrographs from the femoral-patellar and femoral-tibial articulation are depicted in Figure 2K. See Supplemental Figure S1 for the median individual OARSI histopathology indices and composite OA scores.

### 3.3. Articular Cartilage Transcript Expression: PB125 Treatment Had Differing Effects in Males and Females in Genes Related to Nrf2 Signaling in Articular Cartilage (Figure 3)

**Figure 3:**
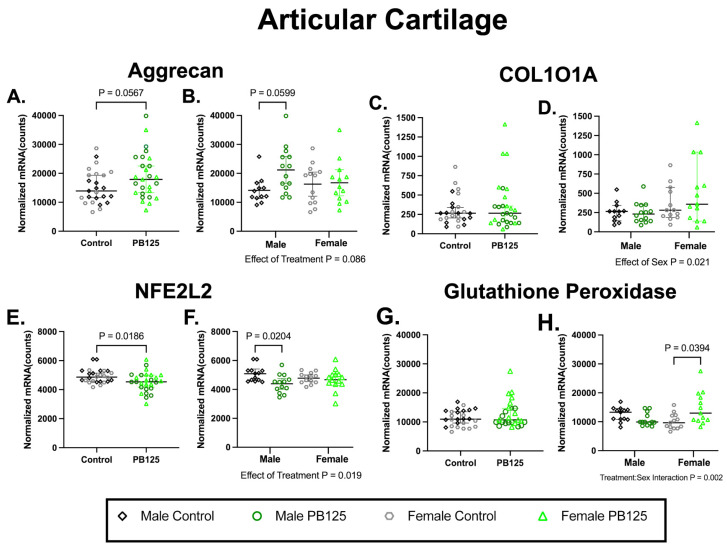
PB125 treatment had differing effects in males and females for transcript expression related to Nrf2 signaling and redox homeostasis in articular cartilage. (**A**,**B**) Analyses did not demonstrate a significant treatment effect in aggrecan expression when sexes were combined or separated. (**C**,**D**) There was a main effect of sex for COL101A; no treatment effects were seen. (**E**,**F**) For NFE2L2, an Nrf2 variant, there was a significant effect of treatment (mean difference: −394 counts) that was driven by male PB125-treated animals (mean difference: −691 counts). (**G**,**H**) PB125-treated females had statistically higher glutathione peroxidase expression (median difference: 3347 counts); a significant treatment/sex interaction was also present.

Aggrecan expression was not statistically affected by PB125 treatment when considering sexes combined or separately (*p* = 0.0567 and *p* = 0.0599; Figure 3A,B, respectively). While a main effect of sex (*p* = 0.021) was present in the collagen type X alpha I chain (COL10A1), no significant treatment differences were detected (Figure 3C,D). Conversely, transcript expression of the Nrf2 variant nuclear factor erythroid-derived 2-like 2 (NFE2L2) was significantly different between treatment groups (*p* = 0.0186; Figure 3E); this appeared to be associated with a significant decrease in transcript expression in male animals (*p* = 0.0204; Figure 3F). While no statistical differences were detected when sexes were combined (Figure 3G), glutathione peroxidase was statistically increased in females receiving PB125 (Figure 3H); a significant treatment/sex interaction (*p* = 0.002) was also present. Please see Supplemental Table S1 for a full list of analyzed genes and descriptive statistics. Collectively, this suggests that PB125 treatment may have altered the transcript expression of an Nrf2 variant in male guinea pigs while targeting a gene related to redox homeostasis in females.

### 3.4. Articular Cartilage IHC: PB125-Treated Guinea Pigs Had Power NQO1 Protein Expression in the Medial Tibial Plateau When Combined for Sex (Figure 4)

NAD(P)H dehydrogenase quinone-1 (NQO1) is a conserved target gene of Nrf2. While in vitro studies demonstrate clear activation of Nrf2 signaling, there are challenges in consistently identifying definitive evidence of Nrf2 activation in vivo. We thus aimed to utilize a downstream mediator of Nrf2 to demonstrate any potential influence of PB125 on this signaling pathway.

**Figure 4:**
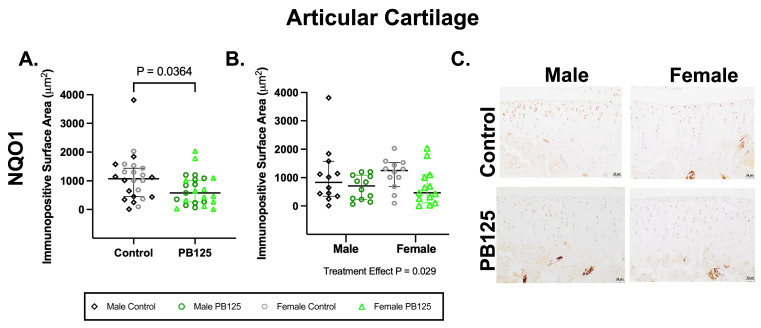
PB125-treated guinea pigs combined for sex had lower NQO1 expression in the medial tibial plateau. (**A**) NQO1 expression was significantly lower in PB125-treated guinea pigs than in controls when animals were combined for sex (median difference: −491.7 μm^2^). (**B**) No significant differences among groups were seen when data was analyzed separately for sex. (**C**) Representative NQO1 IHC photomicrographs of the medial tibial plateau in male (top row), female (bottom row), control (left column), and PB125-treated (right column) Hartley guinea pigs (**B**). 20× objective; bar = 20 μm.

There was a significant effect of PB125 treatment on the medial tibial plateau NQO1 mean immunopositive surface area (*p* = 0.0364, Figure 4A). Specifically, NQO1 immunostaining was statistically lower when considering both males and females treated with PB125. Significant differences were not present when sexes were analyzed separately (Figure 4B). Representative IHC photomicrographs (Figure 4C) demonstrate moderate chondrocyte nuclear and cytoplasmic immunolabeling in the superficial and middle articular zones of control animals. In PB125-treated animals, immunolabeling was more limited to the superficial articular zone.

There was no difference in Nrf2 immunostaining in the medial tibial plateau (Supplemental Figure S2). Because patellar OA scores were intriguing in male guinea pigs treated with PB125 (Figure 2C), NQO1 immunostaining on the patellar surface was quantified; however, no treatment differences were observed (Supplemental Figure S3).

### 3.5. IFP/SC Transcript Expression: PB125 Treatment Significantly Increased Downstream Nrf2 Targets in the IFP (Figure 5)

**Figure 5:**
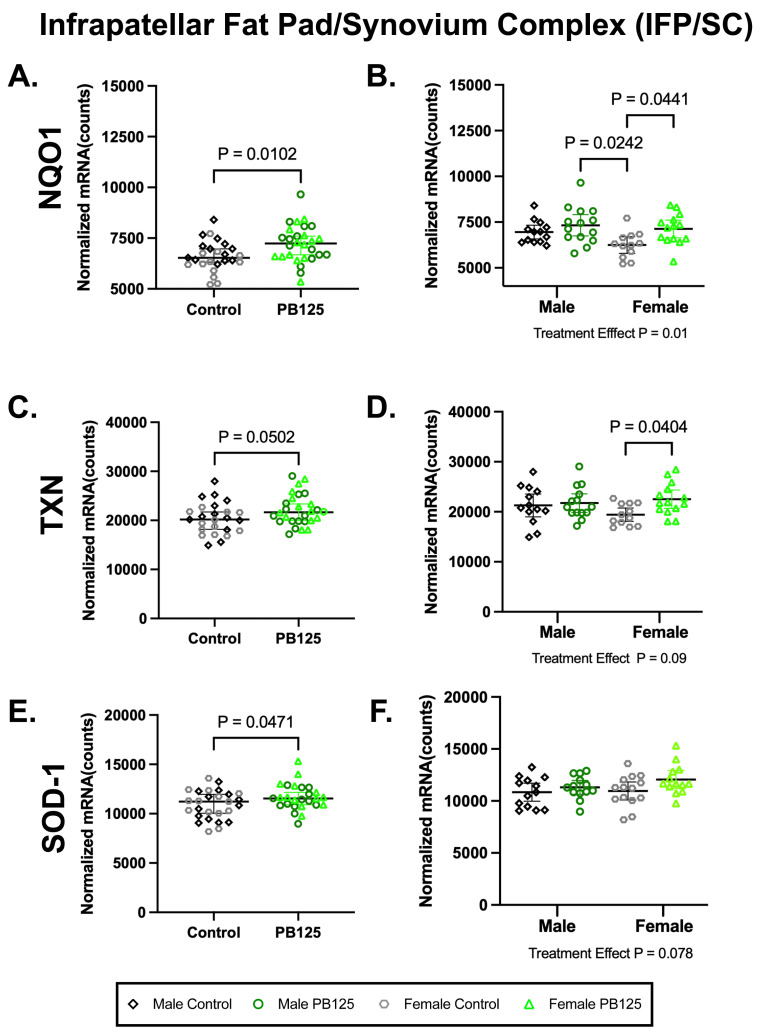
PB125 treatment increased downstream Nrf2 targets in the IPF/SC. (**A**) NQO1 (mean difference: 610 counts) and (**E**) SOD-1 (mean difference: 774 counts) transcript expression were significantly increased by PB125 treatment when sexes were combined. When separated by sex, a statistical difference in female treated animals compared to female controls was found in (**B**) NQO1 (mean difference: 881 counts) but not in (**F**) SOD-1. Near significance (mean difference: 1744 counts) was found in (**C**) combined TXN data; however, a statistical difference (mean difference: 3089 counts) in this transcript (**D**) was present in female guinea pigs when these two groups were considered individually.

There were no effects of treatment on the expression of Nrf2 or NFE2L2 in the IFP/SC in either sex (Supplemental Table S2). There were, however, significant increases in NQO1 (*p* = 0.0102; Figure 5A) and superoxide dismutase-1 (SOD-1) (*p* = 0.0471; Figure 5E) when sexes were combined. NQO1 differences were driven by female treated animals (*p* = 0.0441; Figure 5B), while no statistical group differences were found for SOD-1 (Figure 5F). Thioredoxin (TXN) was not statistically different when data was combined (*p* = 0.0502; Figure 5C) but was significantly higher in PB125-treated females than in the corresponding control group (*p* = 0.0404; Figure 5D). No significant treatment/sex interactions were identified in any transcripts expressed within the IFP. Please see Supplemental Table S2 for a full list of normalized mRNA counts and descriptive statistics from these tissues.

### 3.6. IFP/SC IHC: PB125 Treatment Increased NQO1 Protein Expression When Sexes Were Combined (Figure 6)

**Figure 6:**
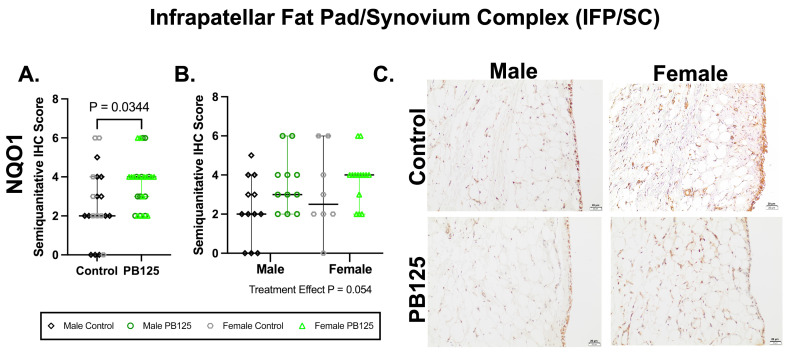
PB125 treatment increased NQO1 protein expression in the IFP/SC when sexes were combined. (**A**) Treatment significantly increased NQO1 when both sexes were considered (median difference: 2.00); (**B**) statistical differences were not detected when groups were analyzed separately. (**C**) Representative NQO1 immunohistochemistry photomicrographs of the IFP/SC from all groups. 20× objective; bar = 20 μm.

There was a significant effect of PB125 treatment on IFP/SC semi-quantitative IHC scores when both sexes were combined (*p* = 0.034, Figure 6A). When separated, no group differences were identified. Representative immunolabeling in synoviocytes, adipocytes, and the fibrovascular interstitium was diminished in PB125-treated animals compared to immunolabeling in controls.

### 3.7. Compulsory and Voluntary Movement: PB125 Treatment Decreased Propel Time in Female Guinea Pigs at 5 Mo (Compulsory); PB125 Decreased the Amount of Time Males Spent in Huts (Voluntary) (Figure 7)

**Figure 7:**
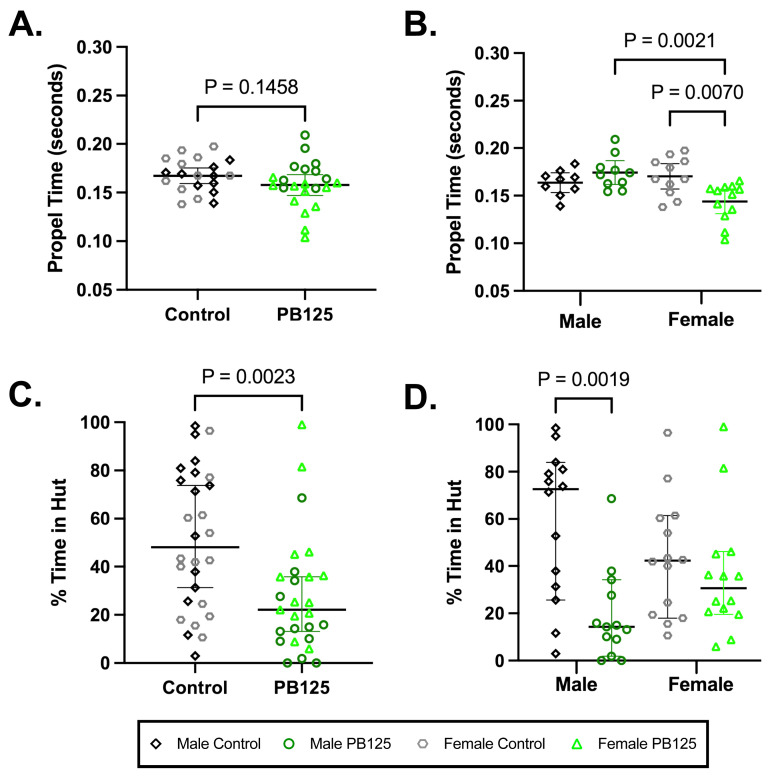
PB125 significantly influenced compulsory and voluntary mobility in Hartley guinea pigs. (**A**) While a comparison of all animals did not reveal a significant difference in propel time, (**B**) female treated animals had a statistically shortened propel time relative to their sex-matched controls (mean difference: −0.0264 s). (**C**) All animals receiving PB125 demonstrated a decrease in the % of time spent in huts (median difference: 25.92%), which was predominantly driven by (**D**) males (median difference: 58.33%).

Compulsory treadmill-based gait analysis did not demonstrate a significant difference when all animals were obligated to run at 55 cm/s (Figure 7A). When groups were divided by sex, however, a significant shortening in propel time was seen in treated females compared to controls (*p* = 0.0070; Figure 7B). No statistically significant differences were found for other parameters; please see Supplemental Table S3 for all indices and descriptive statistics.

Activity levels determined by ANYmaze open field enclosure monitoring, a voluntary measure of movement, revealed that all PB125-treated animals spent significantly less time in huts after 3 months of treatment (*p* = 0.0023; Figure 7C). When investigating the effects of sex, significance was driven by treated males (*p* = 0.0019; Figure 7D). Please see Supplemental Table S4 for all indices and descriptive statistics; Supplemental Figure S4 provides statistics relevant to longitudinal data.

## 4. Discussion

This study investigated the utility of the purported nutraceutical Nrf2 activator, PB125, in modifying the onset of early OA using the translational Hartley guinea pig model of primary OA. As previously mentioned, articular cartilage is naturally of low cellularity with limited renewal capacity and, once damaged, there are essentially no therapeutics available that restore chondrocytes and the extracellular matrix to their native quiescent state. Therefore, in this study, we took the approach of preemptive PB125 treatment before the onset of OA to determine its potential influence on the structural, molecular, and clinical manifestations of early disease in male and female Hartley guinea pigs. In addition to the treatment-related findings, we highlight, below, that there were sex differences in all our outcomes, highlighting the importance of considering treatment effects in both sexes. Our key findings from the present study were that three months of daily oral treatment with PB125 resulted in the following: 1. decreased distal femur OA scores in females, with modified gene and protein expression patterns in both the cartilage and the IFP/SC; and 2. altered clinical (gait and behavior) patterns characteristic of early-stage disease in both males and females.

*PB125 decreased distal femur OA scores in females.* Articular cartilage homeostasis is achieved by a relative balance between anabolic synthesis rates (mediated by growth factors and protease inhibitors) and catalytic degradation (mediated by inflammatory cytokines and proteases). Indeed, when chondrocytes are stimulated through various modes of injury, they often respond through the acquisition of a hypertrophied phenotype and may lose their function in extracellular matrix maintenance and stabilization. Treatment with PB125 did not modify whole knee joint phenotypic manifestations of early OA; however, it did decrease the distal femur scores of femoral-tibial articulation in females.

Correspondingly, we demonstrated altered expression of several Nrf2-induced antioxidants in the articular cartilage and IFP/SC from females. More specifically, articular cartilage exhibited increased glutathione peroxidase mRNA, which may be beneficial in preventing the down-regulation of glutathione peroxidase reported in cartilage from human patients with OA [33]. Furthermore, in the IFP/SC of females treated with PB125, there was increased TXN and NQO1 transcript expression. Thioredoxin, an NADPH oxidase-dependent antioxidant, serves as one of the main control mechanisms for recycling peroxiredoxins, which ultimately mitigates hydrogen peroxide-induced cellular stress. In support of this posit, overexpression of thioredoxins minimized joint destruction in a mouse model of inflammatory osteoarthritis [33,34]. NQO1, an Nrf2-induced quinone reductase, is uniquely relevant, as it can reduce endogenous catechol estrogens generated in estrogen metabolism [35]. Under conditions of oxidative stress, catechol estrogens undergo oxidation and become reactive quinones that drive the Fenton reaction, ultimately releasing toxic iron from ferritin and further perpetuating free radical damage [36,37,38]. Although NQO1 immunoexpression along the medial tibial plateau was decreased with PB125 treatment, PB125-treated females demonstrated increased NQO1 expression in the IFP/SC, which may play a protective role in local mitigation of estrogen-induced oxidative stress within the knee joint. Given these dichotomous differences between articular cartilage and the IFP/SC, future work is necessary to further investigate the effects of PB125 on specific tissue types and determine the potential benefit of decreased versus increased NQO1 expression.

*Key Point 2: PB125 altered clinical (gait and mobility/behavioral) patterns characteristic of early-stage disease in both males and females.* The major presenting complaint in a clinical setting associated with OA is painful and/or decreased mobility [39,40]. Therefore, we sought to characterize a number of functional and clinically relevant outcome measures. Traditional methods utilized to detect pain in rodents include behavioral-based observational scoring systems; however, assessment of these behaviors in guinea pigs can be subtle, non-reproducible, and subjective [41]. In this study, computer-aided gait analysis and overhead enclosure monitoring were used to assess obligatory and voluntary functional mobility, respectively.

Propel time (time after the paw reaches maximum contact area during the stance phase of the stride [I. Mouse Specifics, DigiGait^TM^]) steadily increased during obligatory running (at 55 cm/s) throughout the initial duration of the study (please see Supplemental Table S3). This increase in propel time stabilized around 4 months of age. As animals’ femur lengths, and thus stride lengths, increased, the treadmill belt speed was more easily handled and likely accounted for this steady increase in propel time. Of interest, at 5 months of age, after skeletal maturity has been established, propel time was maintained in control animals but decreased in PB125-treated females. This decrease in propel time may suggest a higher degree of strength and control in the propulsion phase of the gait cycle (I. Mouse Specifics, Digigait). However, as previously stated by Poulet at al., Digigait measurements of brake and propel time have not been validated by force plate analysis, and this is a caveat of this outcome variable [42]. Thus, the biological relevance of this finding requires additional investigation.

When exposed to an open field monitoring system, there were no significant effects of PB125 treatment on total distance traveled, average speed, or time spent mobile (Supplemental Table S4). This was not completely unexpected because, at this early stage of joint degeneration, animals do not always show signs of symptomatic disease. Of note, however, male PB125-treated animals spent significantly less time in their enrichment hut. While the clinical significance of this finding requires further validation, it is plausible that reduced time spent in huts associated with treatment may represent (1) less avoidance of new or threatening places; (2) minimized anxiety-like behavior; and/or (3) differences in nesting behaviors between the sexes [43].

*Caveats.* It is recognized that this study currently provides mostly indirect evidence (e.g., decreased NFE2L2 transcript counts in articular cartilage and NQO1 IHC in two tissue types) that PB125 activated Nrf2 in vivo versus influencing one or more other pathways. Indeed, Nrf2 responses in whole organisms are often transient, cell-type-specific, and highly sensitive to timing and tissue sampling, which may account for the apparent discrepancy between in vitro and in vivo findings. Of note, the animals in the current study were not dosed with PB125 the day of euthanasia. Additional work—particularly involving more work at the protein level, measures of mitochondrial function, additional oxidative damage markers, and downstream signaling pathways in our joint tissue of interest—is needed to determine the exact mechanism(s) by which this dietary supplement may act.

It is also acknowledged that focusing on short-term administration of PB125 in Hartley guinea pigs that are just on the cusp of developing mild primary OA may have reduced the number of statistical differences between groups. This is highlighted in the number of biomarkers, particularly related to pain and inflammation, that were not clearly identified in the manuscript. A longer study in guinea pigs with more advanced disease may better reveal the classic molecules and signaling pathways associated with OA.

Finally, it should be stated that the study attempted to provide a comprehensive approach to determining explanations for the observed differences but that considerations such as hormonal influences and/or differences in individual baseline oxidative stress were not accounted for in the work. Indeed, it is noted that differences in time spent in huts in male PB125-treated guinea pigs, if reflective of their overall activity level, could influence musculoskeletal outcomes independent of PB125. This not only limits the interpretations that can be made from the data but also the translational relevance to humans.

## 5. Conclusions

Collectively, this work revealed that PB125 has the potential to alter several anabolic pathways and antioxidant pathways implicated in OA pathogenesis. Therefore, future longer term investigations are warranted using PB125 to delay the progression of OA in males and females during more advanced end-stage disease in both preclinical and clinical studies.

## Data Availability

The original contributions presented in the study are included in the article. Further inquiries can be directed to the corresponding author.

## Supplementary Materials

The following supporting information can be downloaded at: https://www.mdpi.com/article/10.3390/antiox15020212/s1, Supplemental Table S1: Normalized absolute mRNA counts from pooled articular cartilage and menisci of 5-month-old control and Nrf2-activator treated Hartley guinea pigs. Supplemental Table S2: Normalized absolute mRNA counts from infrapatellar fat pad of 5-month-old control and Nrf2-activator treated guinea pigs. Supplemental Table S3: Any-maze indices depicted as means and standard deviation from 5-month-old control and Nrf2-activator treated guinea pigs. Supplemental Table S4: Digigait indices depicted as means and standard deviation from 5-month-old control and Nrf2-activator treated guinea pigs. Supplemental Figure S1: The median individual OARSI histopathology indices and composite OA scores. Supplemental Figure S2: Nrf2 immunostaining in the medial tibial plateau. Supplemental Figure S3: NQO1 immunostaining on the patellar surface. Supplemental Figure S4: Propel time increased with age while PB125 treatment decreased propel time in female Hartleys at 5 mo and simultaneously increased voluntary movement in males.

## Abbreviations

The following abbreviations are used in this manuscript:

OA	Osteoarthritis
Nrf2	Nuclear factor erythroid 2-related factor-2 (Nrf2)
ARE	Antioxidant response element
ANOVA	Analysis of variance
IFP/SC	Infrapatellar fat pad/synovium complex
IHC	Immunohistochemistry
NQO1	NAD(P)H/quinone oxidoreducatase 1
OARSI	Osteoarthritis and Cartilage Society International

## References

[1] Zhang Y., Jordan J.M. (2010). Epidemiology of osteoarthritis. Clin. Geriatr. Med..

[2] Cisternas M.G., Murphy L., Sacks J.J., Solomon D.H., Pasta D.J., Helmick C.G. (2016). Alternative Methods for Defining Osteoarthritis and the Impact on Estimating Prevalence in a US Population-Based Survey. Arthritis Care Res..

[3] Loeser R.F. (2010). Age-related changes in the musculoskeletal system and the development of osteoarthritis. Clin. Geriatr. Med..

[4] He Y., Li Z., Alexander P.G., Ocasio-Nieves B.D., Yocum L., Lin H., Tuan R.S. (2020). Pathogenesis of Osteoarthritis: Risk Factors, Regulatory Pathways in Chondrocytes, and Experimental Models. Biology.

[5] Pulsatelli L., Addimanda O., Brusi V., Pavloska B., Meliconi R. (2013). New findings in osteoarthritis pathogenesis: Therapeutic implications. Ther. Adv. Chronic Dis..

[6] Bolduc J.A., Collins J.A., Loeser R.F. (2019). Reactive oxygen species, aging and articular cartilage homeostasis. Free Radic. Biol. Med..

[7] Wang L., He C. (2022). Nrf2-mediated anti-inflammatory polarization of macrophages as therapeutic targets for osteoarthritis. Front. Immunol..

[8] Wu K.C., Cui J.Y., Klaassen C.D. (2012). Effect of graded Nrf2 activation on phase-I and -II drug metabolizing enzymes and transporters in mouse liver. PLoS ONE.

[9] Ngo V., Duennwald M.L. (2022). Nrf2 and Oxidative Stress: A General Overview of Mechanisms and Implications in Human Disease. Antioxidants.

[10] Simonds B., Friedman P.J., Sokoloff J. (1975). The prone chest film. Radiology.

[11] Ji L., Li H., Gao P., Shang G., Zhang D.D., Zhang N., Jiang T. (2013). Nrf2 pathway regulates multidrug-resistance-associated protein 1 in small cell lung cancer. PLoS ONE.

[12] Kwak M.K., Itoh K., Yamamoto M., Kensler T.W. (2002). Enhanced expression of the transcription factor Nrf2 by cancer chemopreventive agents: Role of antioxidant response element-like sequences in the nrf2 promoter. Mol. Cell. Biol..

[13] Donovan E.L., McCord J.M., Reuland D.J., Miller B.F., Hamilton K.L. (2012). Phytochemical activation of Nrf2 protects human coronary artery endothelial cells against an oxidative challenge. Oxidative Med. Cell. Longev..

[14] Hwang D.W., Kang J.H., Jeong J.M., Chung J.-K., Lee M.C., Kim S., Lee D.S. (2008). Noninvasive in vivo monitoring of neuronal differentiation using reporter driven by a neuronal promoter. Eur. J. Nucl. Med. Mol. Imaging.

[15] Vargas M.R., Johnson D.A., Sirkis D.W., Messing A., Johnson J.A. (2008). Nrf2 activation in astrocytes protects against neurodegeneration in mouse models of familial amyotrophic lateral sclerosis. J. Neurosci..

[16] Dinkova-Kostova A.T., Kostov R.V., Kazantsev A.G. (2018). The role of Nrf2 signaling in counteracting neurodegenerative diseases. FEBS J..

[17] Davidson R.K., Jupp O., de Ferrars R., Kay C.D., Culley K.L., Norton R., Driscoll C., Vincent T.L., Donell S.T., Bao Y. (2013). Sulforaphane represses matrix-degrading proteases and protects cartilage from destruction in vitro and in vivo. Arthritis Rheum..

[18] Liu L., Tang H., Wang Y. (2023). Polymeric biomaterials: Advanced drug delivery systems in osteoarthritis treatment. Heliyon.

[19] Sun K., Luo J., Jing X., Guo J., Yao X., Hao X., Ye Y., Liang S., Lin J., Wang G. (2019). Astaxanthin protects against osteoarthritis via Nrf2: A guardian of cartilage homeostasis. Aging.

[20] Cai D., Yin S., Yang J., Jiang Q., Cao W. (2015). Histone deacetylase inhibition activates Nrf2 and protects against osteoarthritis. Arthritis Res. Ther..

[21] Musci R.V., Andrie K.M., Walsh M.A., Valenti Z.J., Linden M.A., Afzali M.F., Bork S., Campbell M., Johnson T., Kail T.E. (2023). Phytochemical compound PB125 attenuates skeletal muscle mitochondrial dysfunction and impaired proteostasis in a model of musculoskeletal decline. J. Physiol..

[22] Andrie K.M., Palmer D.R., Wahl O., Bork S., Campbell M., Walsh M.A., Sandord J., Musci R.V., Hamilton K.L., Santangelo K.S. (2024). Treatment with PB125 increases femoral long bone strength in 15-month-old guinea pigs. Ann. Biomed. Eng..

[23] Hybertson B.M., Gao B., Bose S., McCord J.M. (2019). Phytochemical Combination PB125 Activates the Nrf2 Pathway and Induces Cellular Protection against Oxidative Injury. Antioxidants.

[24] Dvir-Ginzberg M., Maatuf Y.H., Mobasheri A. (2024). Do we understand sex-related differences governing dimorphic disease mechanisms in preclinical animal models of osteoarthritis?. Osteoarthr. Cartil..

[25] Strong R., Miller R.A., Antebi A., Astle C.M., Bogue M., Denzel M.S., Fernandez E., Flurkey K., Hamilton K.L., Lamming D.W. (2016). Longer lifespan in male mice treated with a weakly estrogenic agonist, an antioxidant, an α-glucosidase inhibitor or a Nrf2-inducer. Aging Cell.

[26] Bendele A.M., Hulman J.F. (1988). Spontaneous cartilage degeneration in guinea pigs. Arthritis Rheum..

[27] Bendele A.M. (2001). Animal models of osteoarthritis. J. Musculoskel. Neuron. Interact..

[28] Kraus V.B., Huebner J.L., DeGroot J., Bendele A. (2010). The OARSI histopathology initiative—Recommendations for histological assessments of osteoarthritis in the guinea pig. Osteoarthr. Cartil..

[29] Santangelo K., Pieczarka E., Nuovo G., Weisbrode S., Bertone A. (2011). Temporal expression and tissue distribution of interleukin-1β in two strains of guinea pigs with varying propensity for spontaneous knee osteoarthritis. Osteoarthr. Cartil..

[30] Musci R.V., Walsh M.A., Konopka A.R., Wolff C.A., Peelor F.F., Reiser R.F., Santangelo K.S., Hamilton K.L. (2020). The Dunkin Hartley Guinea Pig Is a Model of Primary Osteoarthritis That Also Exhibits Early Onset Myofiber Remodeling That Resembles Human Musculoskeletal Aging. Front. Physiol..

[31] Santangelo K.S., Kaeding A.C., Baker S.A., Bertone A.L. (2014). Quantitative Gait Analysis Detects Significant Differences in Movement between Osteoarthritic and Nonosteoarthritic Guinea Pig Strains before and after Treatment with Flunixin Meglumine. Arthritis.

[32] Sun Y., Scannell B.P., Honeycutt P.R., Mauerhan D.R., H J.N., Hanley E.N. (2015). Cartilage Degeneration, Subchondral Mineral and Meniscal Mineral Densities in Hartley and Strain 13 Guinea Pigs. Open Rheumatol. J..

[33] Yuan Y., Jiao X., Lau W.B., Wang Y., Christopher T.A., Lopez B.L., RamachandraRao S.P., Tao L., Ma X.-L. (2010). Thioredoxin glycation: A novel posttranslational modification that inhibits its antioxidant and organ protective actions. Free Radic. Biol. Med..

[34] Hong C.-C., Ambrosone C.B., Ahn J., Choi J.-Y., McCullough M.L., Stevens V.L., Rodriguez C., Thun M.J., Calle E.E. (2007). Genetic variability in iron-related oxidative stress pathways (Nrf2, NQ01, NOS3, and HO-1), iron intake, and risk of postmenopausal breast cancer. Cancer Epidemiol. Biomark. Prev..

[35] Wyllie S., Liehr J.G. (1997). Release of iron from ferritin storage by redox cycling of stilbene and steroid estrogen metabolites: A mechanism of induction of free radical damage by estrogen. Arch. Biochem. Biophys..

[36] Strong L.C. (1944). Genetic Nature of the Constitutional States of Cancer Susceptibility and Resistance in Mice and Men. Yale J. Biol. Med..

[37] Kasai S., Mimura J., Ozaki T., Itoh K. (2018). Emerging Regulatory Role of Nrf2 in Iron, Heme, and Hemoglobin Metabolism in Physiology and Disease. Front. Vet. Sci..

[38] Burton L.H., Afzali M.F., Radakovich L.B., Campbell M.A., Culver L.A., Olver C.S., Santangelo K.S. (2022). Systemic administration of a pharmacologic iron chelator reduces cartilage lesion development in the Dunkin-Hartley model of primary osteoarthritis. Free Radic. Biol. Med..

[39] Afzali M.F., Pannone S.C., Martinez R.B., Campbell M.A., Sanford J.L., Pezzanite L.M., Kurihara J., Johnson V., Dow S.W., Santangelo K.S. (2023). Intravenous injection of adipose-derived mesenchymal stromal cells benefits gait and inflammation in a spontaneous osteoarthritis model. J. Orthop. Res..

[40] Dieppe P.A., Lohmander L.S. (2005). Pathogenesis and management of pain in osteoarthritis. Lancet.

[41] Ellen Y., Flecknell P., Leach M. (2016). Evaluation of Using Behavioural Changes to Assess Post-Operative Pain in the Guinea Pig (*Cavia porcellus*). PLoS ONE.

[42] Poulet B., de Souza R., Knights C.B., Gentry C., Wilson A.M., Bevan S., Chang Y., Pitsillides A.A. (2014). Modifications of gait as predictors of natural osteoarthritis progression in STR/Ort mice. Arthritis Rheumatol..

[43] Longhi R., Almeida R.F., Pettenuzzo L.F., Souza D.G., Machado L., Quincozes-Santos A., Souza D.O. (2018). Effect of a trans fatty acid-enriched diet on mitochondrial, inflammatory, and oxidative stress parameters in the cortex and hippocampus of Wistar rats. Eur. J. Nutr..

