# Supplementary material for: Three-Month Administration of PB125 Modifies Histopathology, Redox Homeostasis, and Mobility in the Hartley Guinea Pig Model of Primary Osteoarthritis
Source: Antioxidants (Basel). 2026 Feb 5;15(2):212. doi: 10.3390/antiox15020212 (PMC12938315; doi:10.3390/antiox15020212)
Supplement: Supplementary file 1 [file antioxidants-15-00212-s001.zip › Supplemental Figure S4 movement dataLongitudinal data.pdf]

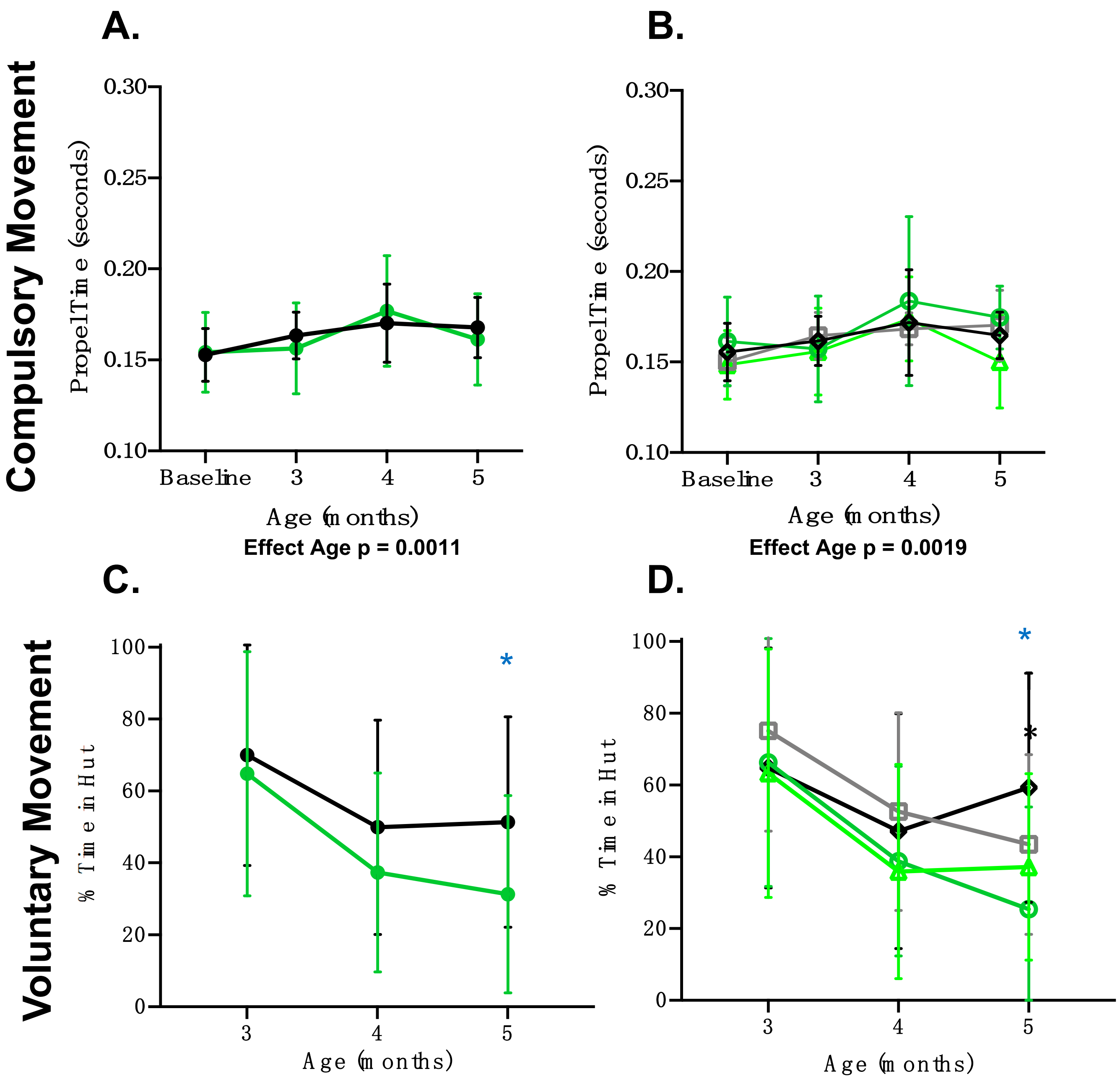

Supplemental Figure S4. *Propel time increased with age while PB125 treatment decreased propel time in female Hartleys at 5 mo and simultaneously increased voluntary movement in males.* (**A**) Longitudinal tracking of mean propel time throughout the study duration in control (black dots and line) and PB125 (green dots and line) treated guinea pigs. A repeated measures mixed effects analysis demonstrated a significant effect of age on mean propel time. (**B**) Longitudinal tracking of individual group mean propel times in male controls (black diamond and line), female controls (gray squares and line), PB125 treated males (green circles and line), and PB125 treated females (neon green triangles and line). A repeated measures mixed effects analysis demonstrated a significant effect of age. (**C**) Longitudinal tracking of the percentage of time spent in hut (%TIH) throughout the study duration in control and PB125 treated guinea pigs ANY-maze™ open field monitoring. A repeated measures 2-way ANOVA demonstrated a significant effect of age and treatment on %TIH. Tukey's post hoc analysis revealed a significant effect of treatment on decreasing %TIH at 5 months. A repeated measures 2-way ANOVA assessing the longitudinal tracking of individual group behavior demonstrated a significant effect of age on %TIH. (**D**) Tukey's post hoc analysis revealed a significant effect of treatment on decreasing %TIH in males at 5 months. **0.01<p<0.05; *** 0.0001<p<0.001*
