# Supplementary material for: Three-Month Administration of PB125 Modifies Histopathology, Redox Homeostasis, and Mobility in the Hartley Guinea Pig Model of Primary Osteoarthritis
Source: Antioxidants (Basel). 2026 Feb 5;15(2):212. doi: 10.3390/antiox15020212 (PMC12938315; doi:10.3390/antiox15020212)
Supplement: Supplementary file 1 [file antioxidants-15-00212-s001.zip › Supplemental Table S1 Nanostring Cart.pdf]

Supplemental Table S1. Normalized absolute mRNA counts from pooled articular cartilage and menisci of 5-month-old control and Nrf2-activator treated Hartley guinea pigs. Data is depicted as mean and standard deviation. Trending (*p<0.15, italic)* and significant (**p<0.05, bold**) sources of variation (determined via 2-way ANOVA, factors signifying sex and treatment) are depicted in table 1. Trending *(p<0.15, italic)* and significant (**p<0.05, bold**) differences between groups (determined via Bonferroni multiple comparisons) are listed in the last column.

2-way ANOVA key:

^{S}*sex*

$^{/}$*treatment*

^{f}*interaction*

NS no significant or trending source of variation identified

Bonferroni multiple comparisons key:

**Difference between control males and PB125 males*

$^{\infty}$*Difference between control females and PB125 females*

$^{\Psi}$*Difference between control males and control females*

$^{\Delta}$*Difference between PB125 males and PB125 females*

NS no significant or trending difference

Gene	Description	Male Control (n=l2)	Male PBl25 (n=l4)	Female Control (n=l3)	Female PBl25 (n=l3)	2-way ANOVA p-values	Bonferroni p-values
**Aggrecan**	Cartilage structure	14177.6; 4450.7	20365.1; 7514.5	16291.7; 6846.1	16977.5; 7380.5	*0.0718*$^{/}$ *0.1471*^{f}	**0.047***
**Adiponectin**	Glucose & FA metabolism	18416.1; 12670.1	11598.8; 5703.4	20295.0; 10224.6	19656.3; 14195.5	*0.1121*^{s}	*0.1288*$^{\Delta}$
**AKT**	Cellular survival and proliferation	6155.7; 1279.8	6144.0; 3997.6	4893.5; 729.5	5779.6; 1869.9	NS	NS
**BAD**	Inducer of apoptosis	1060.2; 172.5	874.0; 89.7	799.0; 111.8	867.2; 177.7	**0.0025**^{t} **0.0016**^{8} *0.1460*$^{/}$	**0.0041*** **<0.0001**$^{\Psi}$
**BAK**	Intrinsic apoptosis	2348.2; 399.9	1970.0; 417.9	1848.1; 297.7	2238.2; 730.3	**0.0075**^{t}	**0.0285**$^{\Psi}$ *0.1205** *0.0967*$^{\infty}$
**BAX**	Intrinsic apoptosis	1474.3; 231.3	1314.1; 158.8	1160.8; 139.5	1378.8; 307.9	**0.0035**^{t} **0.0488**^{8}	**0.0017**$^{\Psi}$ **0.0294**$^{\infty}$ *0.1492**
**BCL-2**	Inhibitor of apoptosis	1510.0; 272.3	1277.2; 333.9	1707.1; 368.6	1499.7; 499.9	**0.0418**^{i} *0.0s20*^{s}	NS
**BECNl**	Regulator of autophagy	2567.5; 416.6	2178.6; 373.9	2021.3; 284.8	2751.6; 1260.3	**0.007l**^{t}	**0.0232**$^{\infty}$ *0.1206*$^{\Psi}$ *0.0898*$^{\Delta}$
**BIM**	Pro-apoptotic BH3 protein	364.4; 56.8	322.4; 58.2	329.1; 67.4	385.9; 145.9	*0.0s77*^{f}	NS

**BMP-7**	Anabolic effect on cartilage	83.7; 20.7	63.2; 20.2	70.9; 18.3	90.1; 17.9	**0.0006**^{f}	**0.02ll***<br>**0.0299**$^{\infty}$<br>**0.00l8**$^{\Delta}$
**C3**	Activator of complement	2224.3; 1108.6	1525.6; 617.9	1680.4; 621.9	2265.2; 1381.1	**0.0224**^{i}	*0.1s19**<br>*0.1113*$^{\Delta}$
**Caspase-l**	Initiator of apoptosis	144.8; 28.6	162.0; 37.2	176.9; 40.2	169.7; 83.7	NS	NS
**Caspase-3**	Executioner of apoptosis	412.5; 57.4	377.4; 52.1	348.2; 62.5	350.5; 79.2	**0.0l28**^{8}	**0.0294**$^{\Psi}$
**Caspase-8**	Executes extrinsic apoptosis	228.3; 23.5	232.9; 78.4	224.4; 33.8	248.3; 58.9	NS	NS
**Caspase-9**	Executes intrinsic apoptosis	277.1; 47.4	221.1; 46.8	218.3; 38.6	266.3; 71.4	**0.00l0**^{t}	**0.02l5***<br>**0.0487**$^{\infty}$<br>**0.0l52**$^{\Psi}$<br>*0.0669*$^{\Delta}$
**Catalase**	Protects from oxidative damage via degradation of $2H_20_2$ ➔ 0_2 + $2H_20$	12115.3; 2026.9	9989.9; 2069.6	13639.4; 2128.4	11808.5; 2044.6	**0.0055**^{8}<br>**0.00l2**^{i}	*0.1438*$^{\Psi}$<br>*0.0s39*$^{\Delta}$
**CBS**	Mediates early transsulfuratio n	37.5; 6.9	34.3; 10.6	35.2; 8.3	37.4; 10.4	NS	NS
**CCL-2**	Chemoattracta nt agent for myeloid and lymphoid cells	3656.6; 1672.1	2892.9; 1574.8	3013.4; 1733.0	2195.7; 1834.6	*0.1014*$^{:j:}$	NS
**CDl63**	Scavenger receptor for hemoglobin-haptoglobin	3025.1; 934.8	2880.2; 513.2	2782.8; 612.9	2360.1; 567.8	**0.0454**^{8}<br>*0.1326*$^{/}$	*0.0976*$^{\Delta}$
**COLl0Al**	Marker of chondrocytes hypertrophy	266.3; 125.5	256.5; 130.1	360.8; 227.1	530.8; 414.1	**0.0ll6**^{8}	*0.0141*$^{\Delta}$
**COL2Al**	Structural component of cartilage	1152287.9; 328219.0	1177245.8; 591386.6	803744.3; 291273.9	998664.2; 268734.5	**0.02l2**^{8}	*0.067s*$^{\Psi}$
**CUL3**	Ubiquitin ligase	3416.3; 167.7	2962.5; 597.4	3392.9; 277.0	3027.3; 681.6	**0.0042**$^{:j:}$	**0.0478***<br>*0.1221*$^{\infty}$
**FGF-l8**	Anabolic effect on cartilage	3367.9; 962.1	3212.8; 1160.9	2760.3; 614.4	2930.1; 840.3	*0.0882*^{s}	NS
**FTH-l**	Intracellular iron storage protein	135133.2; 14696.6	122858.1; 15046.7	128022.6; 14204.5	128593.6; 20333.7	NS	NS
**Glutathione peroxidase**	Nrf2 mediated antioxidant; $2H_20_2$ ➔ 0_2 + $2H_20$<br>$0_2^{=}$ ➔ 0_2 + 0H	12656.4; 2538.3	10727.6; 2286.6	10201.5; 2684.3	15019.2; 5518.2	**0.00l3**^{t}<br>*0.1s08*$^{/}$	**0.0022**$^{\infty}$<br>**0.0065**$^{\Delta}$
**GSK3β**	Inactivates nuclear Nrf2	3029.9; 368.2	2425.6; 433.3	2550.7; 281.6	2417.9; 436.7	**0.0330**^{t}<br>**0.0279**^{8}<br>**0.00l2**^{i}	**0.0005***<br>**0.0065**$^{\Psi}$

**HAMP**	Regulates iron absorption	22.6; 5.8	22.3; 9.6	19.9; 5.9	25.2; 8.2	NS	NS
**HIFl-a**	Transcriptional response to hypoxia	19769.0; 7249.6	19230.3; 6000.9	14781.3; 5331.7	12752.1; 5523.1	**0.0013**[8]	**0.0154**[Δ] *0.0894*[Ψ]
**HMGBl**	Proinflammatory alarmin	15989.9; 2165.9	14522.2; 2265.3	15002.0; 1206.5	14094.7; 2719.9	*0.0s38*[:j:]	NS
**HMOX-l**	Nrf2 regulated antioxidant that degrades heme	2773.6; 907.6	2523.4; 887.9	1973.4; 528.4	2619.0; 1186.3	*0.0849*[f]	*0.06s3*[Ψ]
**IFN-γ**	Implemented in rheumatoid arthritis	11.8; 3.7	14.7; 5.2	11.7; 3.8	15.9; 6.1	*0.0107*[:j:]	*0.0ss6*[∞]
**IL-l0**	Anti-inflammatory cytokine	29.5; 5.9	34.9; 14.7	30.0; 8.1	34.6; 6.7	*0.0717*[:j:]	NS
**IL-lβ**	Proinflammatory cytokine	113.5; 47.9	114.6; 51.8	132.5; 47.8	158.5; 62.4	**0.0373**[8]	*0.0724*[Δ]
**IL-4**	T-cell differentiation and Th2 immune response	69.3; 15.1	68.2; 28.0	56.2; 15.5	59.8; 13.8	**0.0498**[8]	NS
**IL-5**	Induced eosinophil differentiation	27.2; 6.6	30.6; 10.8	26.0; 6.8	26.7; 6.9	NS	NS
**IL-6**	Proinflammatory cytokine	166.6; 92.9	163.7; 81.1	161.6; 156.4	115.8; 48.5	NS	NS
**KEAPl**	Degrades cytoplasmic Nrf2	2127.8; 280.5	2006.5; 521.1	1900.1; 337.5	2455.2; 819.4	**0.0293**[f]	**0.0227**[∞] *0.0767*[Δ]
**LEP**	Adipokine and regulator of appetite	4316.5; 3828.0	2415.9; 3816.8	3325.3; 3989.9	4302.8; 10469.6	NS	NS
**MAPK**	Cell differentiation, proliferation, and survival	4229.8; 393.5	3610.4; 513.6	3741.9; 291.6	3571.8; 672.6	**0.0057**[i] *0.1062*[f] *0.0s96*[s]	**0.0333**[Ψ] **0.0048**[*]
**MCLl**	Inhibitor of apoptosis	8323.5; 849.5	7284.9; 925.1	7497.6; 639.7	7118.5; 1253.8	*0.0647*[s] **0.0095**[i]	*0.0678*[Ψ] **0.0149**[*]
**MMP-l3**	Cleaves type II collagen	5182.3; 2267.4	4999.4; 1857.7	5879.9; 2153.6	7074.5; 3052.9	**0.0402**[8]	*0.0s47*[Δ]
**MMP-2**	Cleaves type IV collagen	61945.9; 12277.3	64706.1; 23118.5	48541.4; 8796.2	48396.6; 13700.5	**0.0013**[s]	**0.0190**[Δ] *0.07ss*[Ψ]
**MMP-3**	Cleaves type II-IV, IX and X collagens	760.2; 165.0	717.3; 154.5	691.3; 198.0	624.5; 211.9	*0.1197*[s]	NS
**MMP-9**	Cleaves type IV & V collagen, activates neutrophils	3709.7; 1374.7	3430.6; 1585.7	3553.5; 1136.3	5149.5; 1586.0	**0.0230**[f] *0.0s62*[s] *0.10s6*[/]	**0.0064**[Δ] **0.0135**[∞]
**MTOR**	Regulator of cell metabolism and growth	1356.8; 342.1	978.2; 180.9	1012.9; 236.9	1270.3; 599.7	**0.0042**[f]	**0.0311**[*] *0.0s43*[Ψ] *0.1079*[Δ]

**NF-κB-l**	Proinflammatory transcription factor	488.6; 68.3	448.2; 45.9	459.6; 46.4	469.5; 92.6	NS	NS
**NFE-2**	Oxidant induced anti-inflammatory transcription factor	450.6; 346.5	405.5; 196.2	605.4; 369.6	636.5; 400.6	NS	NS
**NFE2L2**	Oxidant induced anti-inflammatory transcription factor	5140.5; 540.1	4475.7; 566.0	4803.4; 357.5	4737.1; 703.4	**0.02l6**^{i} *0.0s78*^{f}	**0.0080***
**NOSl**	Neuronal and constitutively expressed; synthesizes NO	73.8; 21.4	70.7; 30.1	61.7; 17.2	75.1; 22.3	NS	NS
**NOS2**	Cytokine induced NO synthesis	423.1; 116.0	376.9; 138.6	329.1; 105.0	389.9; 104.3	*0.108s*^{f}	*0.1002*$^{\Psi}$
**NOS3**	Endothelial induced NO in shear stress	143.9; 59.4	114.0; 31.4	103.2; 30.6	134.7; 75.9	**0.0435**^{t}	*0.1210*$^{\Psi}$
**NQOl**	Nrf2 mediated removal of toxic intermediates	6966.7; 832.9	6897.3; 932.6	5978.1; 740.7	6267.6; 701.9	**0.0008**^{8}	**0.0074**$^{\Psi}$ *0.0979*$^{\Delta}$
**NURRl**	Neuroinflammatory transcription factor	2492.3; 1122.8	1841.2; 654.0	1778.9; 864.1	1907.1; 814.9	**0.l098**^{t}	*0.0914*$^{\Psi}$ *0.12s6**
**Nrfl**	Transcriptional modulation of metabolism, growth, and development	505.6; 55.4	497.1; 51.1	544.5; 57.2	564.9; 75.6	**0.0026**^{8}	**0.0l08**$^{\Delta}$
**PCSK9**	LDL-cholesterol metabolism	17.0; 7.0	13.8; 2.8	11.5; 3.0	16.4; 22.0	NS	NS
**PPARγ**	Adipocyte differentiation; senescence	480.4; 230.7	330.4; 82.5	443.6; 135.6	491.4; 201.1	**0.0400**^{t}	**0.03l5**$^{\Delta}$ *0.0s9s**
**PRDXl**	Nrf2 antioxidant; detoxifies peroxides	10976.2; 1277.7	10803.7; 1444.5	9970.1; 1208.4	8545.8; 2649.9	**0.00l8**^{8} *0.1112*$^{/}$	
**PTGS-l**	Proinflammatory enzyme	703.6; 145.5	761.5; 492.9	630.2; 122.1	1000.4; 953.4	NS	NS
**PTGS-2**	Proinflammatory enzyme	179.1; 108.7	165.3; 73.1	168.5; 108.1	153.0; 102.0	NS	NS
**RIPKl**	Driver of apoptosis and necroptosis	1581.3; 113.7	1499.5; 145.1	1517.7; 117.7	1400.0; 247.9	**0.0359**^{i} *0.0840*^{s}	*0.1499*$^{\infty}$
**RUNX2**	Osteoblast differentiation	1210.9; 261.8	1024.9; 260.5	1156.2; 263.2	1345.9; 389.3	**0.0278**^{t} *0.114s*^{s}	**0.0l38**$^{\Delta}$

**SCLllA2**	Metal transport, iron uptake	1376.7; 158.3	1414.5; 376.2	1252.4; 193.8	1607.2; 768.5	*0.12s3*[:j:]	*0.0989*[∞]
**SESN2**	Stress-induced metabolic regulator	146.4; 35.9	115.0; 23.8	122.2; 37.7	139.4; 58.7	**0.0401**[t]	*0.1246*[*]
**SLC39A14**	Divalent metal transporter	1607.1; 726.4	1522.4; 547.6	1158.3; 459.8	1532.4; 1166.9	NS	NS
**SLC40A1**	Ferroportin; transports iron	2373.8; 443.4	2425.2; 286.2	2740.7; 380.1	2354.4; 456.2	*0.130s*[:j:]<br>*0.0s00*[f]	**0.0494**[Ψ]<br>**0.0289**[∞]
**SLC7A11**	Cystine/gluta mate antiporter; preserves redox balance	76.6; 19.9	77.4; 17.6	57.3; 18.3	71.0; 44.0	*0.0992*[s]	NS
**SOD-1**	Cytoplasmic dismutation of $O_2^- \rightarrow O_2$ + H_2O_2	9109.6; 401.6	8899.6; 876.4	8818.8; 768.3	9287.7; 1228.2	NS	NS
**SOD-2**	Mitochondrial dismutation of $O_2^- \rightarrow O_2$ + H_2O_2	21789.7; 9020.4	19333.1; 4950.9	19101.2; 9289.0	15365.0; 7151.7	0.1234[s]	NS
**SOD-3**	Extracellular dismutation of $O_2^- \rightarrow O_2$ + H_2O_2	3950.4; 1100.8	3058.8; 1140.5	3239.4; 1017.3	4364.9; 2214.7	**0.0171**[t]	*0.0s63*[Δ]<br>*0.1138*[∞]
**TFRC**	Transferrin receptor; cellular uptake of iron	1677.2; 512.3	1540.0; 427.4	1805.2; 653.0	1824.8; 713.2	NS	NS
**TGFβ1**	Secretory peptide; cell growth, differentiation , and apoptosis	2017.3; 245.2	1644.9; 294.4	1661.0; 248.8	2079.7; 570.5	**0.0004**[t]	**0.0296**[*]<br>**0.0112**[∞]<br>**0.0388**[Ψ]<br>**0.0083**[Δ]
**TIMP-1**	Inhibits ECM degradation	8450.0; 2482.7	7402.1; 1670.5	6875.4; 1852.8	8027.1; 3270.6	*0.1087*[f]	NS
**TIMP-2**	Inhibits ECM degradation	42795.2; 6818.1	41424.2; 6636.4	38960.4; 5591.7	36823.3; 8335.2	**0.0346**[8]	NS
**TNF**	Proinflammat ory cytokine involved in the acute phase stress response	30.1; 6.9	27.4; 8.9	28.3; 6.6	31.7; 11.5	NS	NS
**TXN**	Nrf2 induced antioxidant; reduces oxidized cysteine residues and cleaves disulfide bonds	15090.5; 2635.8	13431.1; 3251.7	12606.9; 1945.5	11696.3; 4446.8	**0.0223**[8]	*0.1237*[Ψ]
**ULK1**	Inducer of autophagy	1577.2; 174.1	1362.6; 304.2	1469.7; 250.9	1709.8; 571.5	**0.0292**[t]	**0.0358**[Δ]

**VEGF**	Promotes growth of new blood vessels	12.2; 5.1	12.5; 4.9	13.3; 3.7	14.0; 6.7	NS	NS
**WNT**	Regulates the proliferation of cells	15.0; 5.3	16.1; 9.5	13.4; 6.2	24.3; 10.0	**0.0358**[t]<br>**0.0102**[i]<br>*0.1497*[s]	**0.0238**[Δ]<br>**0.0025**[∞]
**β-CAT**	Component of canonical Wnt signaling; cell growth and adhesion	24800.6; 3794.0	21997.0; 1954.2	20918.3; 2232.7	24234.3; 4831.0	**0.0024**[t]	**0.0129**[Ψ]<br>**0.0332**[∞]<br>*0.0902*[*]
**p65 {RELA)**	Nuclear translocation and activation of NF-κB	5800.5; 957.0	4768.1; 727.7	4800.6; 660.5	5462.4; 1667.0	**0.0075**[t]	***0.0424***[*]<br>*0.0s08*[Ψ]
**ACTB**	Housekeeper gene; constitutively expressed cytoskeletal protein	167191.7; 19438.2	146372.2; 11088.5	145915.8; 18080.0	148469.0; 24220.3	**0.0284**[t]<br>*0.0698*[s]<br>*0.0837*[:j:]	**0.0135**[Ψ]<br>**0.0140**[*]
**EEFlal**[&]	Housekeeper gene; delivery of tRNAs to the ribosome	171648.9; 0.0	171648.9; 0.0	171648.9; 0.0	171648.9; 0.0	*0.112s*[f]	NS
**GAPDH**	Housekeeper gene; catalyzes the 6th step of glycolysis	24722.3; 6993.9	27926.1; 16914.3	22352.4; 7152.2	32476.4	NS	NS
**SDH-a**	Housekeeper gene; involved in complex II of the mitochondrial electron transport chain	1886.4; 215.3	2260.0; 1082.6	2004.5; 173.7	2616.3; 1933.4	*0.12s8*[f]	NS

[&]*Selected housekeeper gene utilized in data normalization*
