# Supplementary material for: Three-Month Administration of PB125 Modifies Histopathology, Redox Homeostasis, and Mobility in the Hartley Guinea Pig Model of Primary Osteoarthritis
Source: Antioxidants (Basel). 2026 Feb 5;15(2):212. doi: 10.3390/antiox15020212 (PMC12938315; doi:10.3390/antiox15020212)
Supplement: Supplementary file 1 [file antioxidants-15-00212-s001.zip › Supplemental Table S2.Normalized mRNA counts IFP_SC.pdf]

Supplemental Table S2. Normalized absolute mRNA counts from infrapatellar fat pad of 5-month-old control and Nrf2-activator treated guinea pigs. Data is depicted as mean and standard deviation. Trending *(p<0.15, italic)* and significant (**p<0.05, bold**) sources of variation (determined via 2-way ANOVA, factors signifying sex and treatment) are depicted in table 1. Trending *(p<0.15, italic)* and significant (**p<0.05, bold**) differences between groups (determined via Bonferroni multiple comparisons) are listed in the last column.

2-way ANOVA key:

^{S}sex
$^{/}$treatment
^{f}interaction
NS no significant or trending source of variation identified

Bonferroni multiple comparisons key:

*Difference between control males and PB125 males
$^{\infty}$Difference between control females and PB125 females
$^{\Psi}$Difference between control males and control females
$^{\Delta}$Difference between PB125 males and PB125 females
NS no significant or trending difference

Gene	Description	Male Control (n=l3)	Male PBl25 (n=l4)	Female Control (n=l3)	Female PBl25 (n=l4)	2-way ANOVA p-values	Bonferroni p-values
**Aggrecan**	Cartilage structure	184.1; 98.3	207.3; 129.9	184.6; 144.9	154.2; 40.9	NS	NS
**Adiponectin**	Glucose & FA metabolism	107502.9; 29708.8	101251.7; 27382.9	143535.8; 36358.4	130632.4; 28836.2	**0.0003^{8}**	**0.0085$^{\Psi}$ 0.0288$^{\Delta}$**
**AKT**	Cellular survival and proliferation	2942.5; 530.4	3060.1; 610.4	2805.6; 448.3	2776.8; 451.7	*0.1408^{S}*	NS
**BAD**	Inducer of apoptosis	777.6; 102.7	807.8; 115.0	722.3; 126.3	719.7; 80.7	**0.0l76^{8}**	*0.0690$^{\Delta}$*
**BAK**	Intrinsic apoptosis	1545.3; 251.3	1616.5; 340.3	1371.8; 226.2	1430.8; 257.9	**0.0l97^{8}**	NS
**BAX**	Intrinsic apoptosis	833.0, 106.4	827.6; 132.7	758.4; 136.2	739.7; 123.2	**0.02ll^{8}**	*0.1384$^{\Delta}$*
**BCL-2**	Inhibitor of apoptosis	1160.1; 223.1	1265.8; 257.8	1309.2; 276.4	1485.3; 293.9	**0.0l36^{8}** *0.0s62$^{/}$*	*0.06s8$^{\Delta}$*
**BECNl**	Regulator of autophagy	1642.5; 239.8	1632.1; 308.6	1555.5; 198.9	1528.7; 141.4	*0.1361^{S}*	NS
**BIM**	Pro-apoptotic	324.7; 73.7	336.8; 79.2	351.0; 124.2	284.1; 36.4	0.0873^{f}	0.0853$^{\infty}$
**BMP-7**	Anabolic effect on cartilage	67.7; 43.4	68.1; 46.6	64.5; 45.4	56.7; 22.4	NS	NS
**C3**	Activator of complement	5784.7; 1930.1	4795.1; 1001.8	6241.1; 1824.4	5470.7; 1287.1	**0.04l5^{i}**	NS
**Caspase-l**	Initiator of apoptosis	487.7; 111.0	492.5; 159.7	375.8; 102.2	405.8; 89.5	**0.0035^{8}**	**0.0406$^{\Psi}$** *0.1199$^{\Delta}$*
**Caspase-3**	Executioner of apoptosis	510.3; 95.2	533.8; 152.3	457.6; 169.9	455.6; 73.3	*0.0674^{S}*	NS

**Caspase-8**	Executes extrinsic apoptosis	313.5; 34.8	307.6; 37.8	299.6; 54.3	292.5; 29.5	NS	NS
**Caspase-9**	Executes intrinsic apoptosis	188.8; 37.3	181.4; 29.2	186.6; 46.7	178.3; 24.1	NS	NS
**Catalase**	Protects from oxidative damage via degradation of $2H_20_2 \rightarrow 0_2{+}2H_20$	27085.3; 4991.0	23187.3; 4993.1	31188.1; 5865.2	29816.9; 4430.6	**0.0003**^{8} *0.0627*$^{/}$	**0.0023**$^{\Delta}$ *0.1038**
**CBS**	Mediates early transsulfuration	62.4; 15.2	69.6; 31.9	71.2; 15.7	66.6; 11.4	NS	NS
**CCL-2**	Chemoattractant agent for myeloid and lymphoid cells	4165.7; 2363.9	3752.4; 1614.8	3312.4; 1178.4	3940.8; 1674.2	NS	NS
**CD163**	Scavenger receptor for hemoglobin-haptoglobin	8374.6; 1542.4	9079.9; 2534.8	7002.1; 1535.0	7797.0; 1881.9	**0.0148**^{8}	NS
**COL10A1**	Marker of chondrocytes hypertrophy	94.7; 72.0	100.7; 108.9	98.3; 95.1	72.2; 37.4	NS	NS
**COL2A1**	Structural component of cartilage	431.7; 463.8	687.4; 553.7	251.5; 133.9	700.0; 611.3	**0.0104**^{i}	**0.0385**$^{\infty}$
**CUL3**	Ubiquitin ligase	34889.0; 336.3	3629.4; 440.0	3205.7; 239.2	3457.0; 281.8	**0.0157**^{8} **0.0364**^{t}	*0.0713*$^{\Psi}$ *0.1133*$^{\infty}$
**FGF-18**	Anabolic effect on cartilage	2013.4; 792.5	1947.0; 365.6	2334.0; 740.7	2268.5; 672.4	*0.0799*^{S}	NS
**FTH-1**	Intracellular iron storage protein	105628.4; 18656.3	102444.2; 12637.8	101435.9; 13217.6	100003.3; 10091.8	NS	NS
**Glutathione peroxidase**	Nrf2 mediated antioxidant; $2H_20_2 \rightarrow 0_2 + 2H_20$ $0_2^{=} \rightarrow 0_2 + 0H$	6321.7; 1261.5	6380.0; 1138.7	6232.6; 1608.1	6224.1; 1120.2	NS	NS
**GSK3β**	Inactivates nuclear Nrf2	2076.3; 128.6	2187.5; 201.8	1957.9; 254.9	2084.8; 167.4	**0.0410**^{8} **0.0283**^{i}	NS
**HAMP**	Regulates iron absorption	98.3; 61.4	91.1; 73.3	97.1; 73.4	78.4; 29.4	NS	NS
**HIF1-a**	Transcriptional response to hypoxia	6719.3; 1882.2	8389.3; 2811.2	5841.1; 1194.6	5736.1; 1209.6	**0.0013**^{8} *0.0933*^{f} *0.1377*$^{:t}$	**0.0543*** **0.0011**$^{\Delta}$
**HMGB1**	Proinflammatory alarmin	15477.0; 1495.0	15444.7; 1349.8	13922.9; 990.8	14924.2; 916.1	**0.0028**^{8} *0.1231*^{f} *0.1478*$^{/}$	**0.0038**$^{\Psi}$ *0.0731*$^{\infty}$
**HMOX-1**	Nrf2 regulated antioxidant that degrades heme	1600.7; 391.9	1526.2; 347.0	1374.8; 440.9	1216.8; 322.7	**0.0134**^{8}	*0.0787*$^{\Delta}$
**IFN-γ**	Implemented in rheumatoid arthritis	33.0; 20.6	34.1; 25.1	32.9; 26.5	31.4; 13.5	NS	NS
**IL-10**	Anti-inflammatory cytokine	103.9; 46.4	108.5; 55.8	97.1; 52.9	90.9; 20.1	NS	NS
**IL-1β**	Proinflammatory cytokine	177.1; 61.0	163.5; 86.0	139.2; 42.7	143.0; 30.5	*0.0761*^{s}	NS
**IL-4**	T-cell differentiation and	67.9; 43.4	69.1; 59.4	66.5; 45.5	52.6; 15.0	NS	NS

	Th2 immune response						
**IL-5**	Induced eosinophil differentiation	43.0; 16.2	44.3; 19.9	47.3; 24.0	43.2; 13.4	NS	NS
**IL-6**	Proinflammatory cytokine	267.7; 147.3	206.2; 140.1	182.5; 146.7	131.6; 41.5	**0.0242**[8]<br>*0.1084*[:t]	NS
**KEAPl**	Degrades cytoplasmic Nrf2	1234.3; 219.7	1294.0; 242.2	1192.6; 220.3	1217.1; 201.0	NS	NS
**LEP**	Adipokine and regulator of appetite	71135.1; 38306.7	69985.0; 40246.5	61065.7; 20423.6	47909.3; 16989.8	*0.0611*[s]	*0.1274*[Δ]
**MAPK**	Cell differentiation, proliferation, and survival	3199.4; 360.2	3404.1; 284.3	2996.7; 311.6	3311.3; 292.3	**0.0036**[i]<br>*0.0887*[s]	**0.0236**[∞]
**MCLl**	Inhibitor of apoptosis	9922.9; 1027.0	9438.1; 1020.1	8792.3; 1185.1	8822.4; 1211.9	**0.0059**[8]	**0.0253**[Ψ]
**MMP-l3**	Cleaves type II collagen	106.6; 61.5	112.7; 53.8	116.2; 88.6	106.0; 47.8	NS	NS
**MMP-2**	Cleaves type IV collagen	23817.8; 5723.6	28484.9; 9573.6	22754.2; 5190.8	20932.2; 4958.9	**0.02l7**[8]<br>*0.0804*[f]	**0.0085**[Δ]
**MMP-3**	Cleaves type II-IV, IX and X collagens	456.2; 117.4	415.7; 87.1	318.8; 79.8	273.9; 79.2	**<0.000l**[8]<br>*0.0984*[:t]	**0.0008**[Ψ]<br>**0.0004**[Δ]
**MMP-9**	Cleaves type IV & V collagen, activates neutrophils	59.0; 26.1	62.6; 27.5	64.8; 46.1	57.9; 36.9	NS	NS
**MTOR**	Cell metabolism and growth	529.6; 106.7	543.2; 152.9	496.5; 108.2	462.6; 70.6	*0.0722*[s]	*0.1327*[Δ]
**NF-κB-l**	Proinflammatory transcription factor	337.3; 46.7	338.1; 43.7	313.7; 62.5	318.4; 45.7	*0.1179*[s]	NS
**NFE-2**	0xidant induced anti-inflammatory transcription factor	144.2; 29.0	133.9; 22.4	146.6; 35.0	153.8; 34.5	NS	NS
**NFE2L2**	0xidant induced anti-inflammatory transcription factor	4880.3; 453.1	4969.5; 394.9	4624.5; 533.2	4855.8; 347.4	*0.12s4*[s]	NS
**NOSl**	Neuronal and constitutively expressed; synthesizes N0	27.8; 19.0	36.0; 27.1	23.8; 14.2	27.9; 33.4	NS	NS
**NOS2**	Cytokine induced N0 synthesis	157.7; 99.2	166.4; 144.9	150.4; 115.7	116.8; 54.4	NS	NS
**NOS3**	Endothelial induced N0 in shear stress	135.3; 34.9	123.5; 28.6	133.5; 36.9	112.7; 24.2	*0.0626*[:t]	NS
**NQOl**	Nrf2 mediated removal of toxic intermediates	6980.9; 620.0	7331.1; 1007.0	6283.8; 637.3	7063.7; 818.1	**0.0299**[8]<br>**0.0116**[i]	**0.0273**[∞]<br>*0.0s86*[Ψ]
**NURRl**	Neuroinflammatory transcription factor	1405.8; 782.1	915.0; 230.2	827.2; 297.4	833.6; 405.6	**0.0l36**[8]<br>*0.0664*[/]<br>*0.0s98*[f]	**0.006l**[Ψ]<br>**0.0l95**[*]
**Nrfl**	Transcriptional modulation of metabolism,	515.3; 44.3	510.4; 64.1	488.6; 81.9	507.7; 51.2	NS	NS

	growth, and development						
**PCSK9**	LDL-cholesterol metabolism	56.4; 37.6	47.6; 40.4	45.5; 32.9	37.8; 14.9	NS	NS
**PPARγ**	Adipocyte differentiation; senescence	1834.3; 473.4	1616.4; 463.8	2107.6; 503.6	1986.3; 601.2	**0.0259**^{8}	*0.12s6*$^{\Delta}$
**PRDXl**	Nrf2 antioxidant; detoxifies peroxides	14163.4; 2077.5	14550.4; 1606.0	12950.4; 1490.2	14374.8; 1578.5	*0.0ss9*$^{:t}$ *0.1399*^{s}	*0.149s*$^{\Psi}$ *0.0686*$^{\infty}$
**PTGS-l**	Proinflammatory enzyme	1044.1; 201.3	1018.5; 174.6	927.5; 194.1	945.7; 160.0	*0.0628*^{s}	NS
**PTGS-2**	Proinflammatory enzyme	220.9; 86.5	189.9; 90.3	192.1; 116.6	172.0; 47.0	NS	NS
**RIPKl**	Driver of apoptosis and necroptosis	1674.7; 145.0	1723.5; 186.1	1530.2; 211.0	1610.2; 139.6	**0.0085**^{8}	*0.07ss*$^{\Psi}$
**RUNX2**	0steoblast differentiation	109.8; 38.9	118.6; 43.1	102.7; 57.9	84.9; 18.8	*0.0784*^{S}	*0.07s2*$^{\Delta}$
**SCLllA2**	Metal transport, iron uptake	902.9; 140.9	949.6; 195.6	866.0; 235.9	829.1; 82.4	*0.100s*^{S}	*0.1417*$^{\Delta}$
**SESN2**	Stress-induced metabolic regulator	55.2; 11.7	58.4; 13.2	50.0; 12.6	47.9; 8.6	**0.0l79**^{8}	**0.0434**$^{\Delta}$
**SLC39Al4**	Divalent metal transporter	397.9; 206.4	423.9; 254.8	335.8; 165.8	353.6; 217.8	NS	NS
**SLC40Al**	Ferroportin; transports iron	2791.5; 548.5	2820.0; 497.7	2835.4; 472.8	2657.8; 358.0	NS	NS
**SLC7All**	Cystine/glutamate antiporter; preserves redox balance	80.7; 40.1	94.6; 35.2	52.2; 21.9	62.3; 29.9	**0.00l2**^{8}	*0.0600*$^{\Psi}$ **0.0227**$^{\Delta}$
**SOD-l**	Cytoplasmic dismutation of $0_2^- \rightarrow 0_2 + H_2 0_2$	10854.7; 1416.3	11361.1; 997.4	10906.5; 1276.8	12065.3; 1495.2	**0.0235**^{i}	*0.0s14*$^{\infty}$
**SOD-2**	Mitochondrial dismutation of $0_2^- \rightarrow 0_2 + H_2 0_2$	14718.4; 5601.6	15816.6; 5787.9	12198.1; 3025.7	12008.4; 2917.2	**0.0l36**^{8}	*0.0624*$^{\Delta}$
**SOD-3**	Extracellular dismutation of $0_2^- \rightarrow 0_2 + H_2 0_2$	1123.9; 229.5	1041.3; 259.6	1351.1; 379.8	1193.8; 268.0	**0.0l95**^{8} *0.1337*$^{:t}$	*0.1007*$^{\Psi}$
**TFRC**	Transferrin receptor; cellular uptake of iron	654.4; 104.3	678.4; 174.5	610.5; 157.4	596.1; 91.9	*0.0964*^{s}	NS
**TGFβl**	Secretory peptide; cell growth, differentiation, and apoptosis	789.5; 190.9	824.7; 241.4	751.7; 120.7	826.8; 164.5	NS	NS
**TIMP-l**	Inhibits ECM degradation	5512.5; 1942.0	5016.6; 1993.0	4615.2; 2435.7	4028.5; 1279.7	*0.0814*^{s}	NS
**TIMP-2**	Inhibits ECM degradation	43302.4; 3924.0	44454.8; 6146.0	42844; 7046.9	41875.2; 5433.6	NS	NS
**TNF**	Proinflammatory cytokine involved in the acute phase stress response	57.7; 19.7	55.3; 29.7	52.6; 27.7	45.7; 9.8	NS	NS

**TXN**	Nrf2 induced antioxidant; reduces oxidized cysteine residues and cleaves disulfide bonds	21391.4; 3790.7	21770.3; 3139.9	19038.5; 1732.1	22511.3; 3242.0	**0.0258**$^{\mathbf{i}}$ *0.0708*^{f}	**0.0102**$^{\infty}$ *0.1136*$^{\Psi}$
**ULKl**	Inducer of autophagy	790.2; 133.7	827.8; 198.6	774.6; 130.4	759.4; 102.4	NS	NS
**VEGF**	Promotes growth of new blood vessels	50.2; 35.9	53.9; 63.8	46.1; 31.6	45.1; 18.7	NS	NS
**WNT**	Regulates the proliferation of cells	91.1; 70.3	71.7; 53.2	88.0; 79.1	68.3; 31.0	NS	NS
**β-CAT**	Component of canonical Wnt signaling; cell growth and adhesion	9730.2; 1062.5	10418.6; 1979.5	9526.8; 1752.2	9328.6; 1383.2	*0.1413*^{s}	NS
**p65 (RELA)**	Nuclear translocation and activation of NF-κB	2616.0; 555.3	2682.3; 540.8	2553.0; 542.8	2492.0; 477.6	NS	NS
**ACTB**	Housekeeper gene; constitutively expressed cytoskeletal protein	95417.7; 14725.3	95812.3; 21297.0	85734.4; 20222.5	87110.7; 21259.8	*0.0918*^{s}	NS
**EEFlal**[&]	Housekeeper gene; delivery of tRNAs to the ribosome	171648.9; 0.0	171648.9; 0.0	171648.9; 0.0	171648.9; 0.0	NS	NS
**GAPDH**	Housekeeper gene; catalyzes the 6th step of glycolysis	8660.6; 1603.0	10946.8; 6462.6	8102.7; 1992.5	9261.6; 3698.2	*0.1200*$^{:t}$	NS
**SDH-a**	Housekeeper gene; involved in complex II of the mitochondrial electron transport chain	2912.2; 258.5	2958.9; 430.3	2920.0; 330.2	2864.0; 314.1	NS	NS

[&]Selected housekeeper gene utilized in data normalization
