# Supplementary material for: Three-Month Administration of PB125 Modifies Histopathology, Redox Homeostasis, and Mobility in the Hartley Guinea Pig Model of Primary Osteoarthritis
Source: Antioxidants (Basel). 2026 Feb 5;15(2):212. doi: 10.3390/antiox15020212 (PMC12938315; doi:10.3390/antiox15020212)
Supplement: Supplementary file 1 [file antioxidants-15-00212-s001.zip › Supplemental Table S3 AnyMaze .pdf]

Supplemental Table S3. Any-maze indices depicted as means and standard deviation from 5-month- old control and Nrf2-activator treated guinea pigs. Trending *(p<0.15, italic)* and significant (**p<0.05, bold**) sources of variation (determined via 2-way ANOVA, factors signifying sex and treatment) are shown. Trending *(p<0.15, italic)* and significant (**p<0.05, bold**) differences between groups (determined via Bonferroni multiple comparisons) are listed in the last column.

2-way ANOVA key:

[S]sex
[I]treatment
[f]interaction[f]
NS no significant or trending source of variation identified

Bonferroni multiple comparisons key:

[*]Difference between control males and PB125 males
[∞]Difference between control females and PB125 females [Ψ]Difference between control males and control females [Δ]Difference between PB125 males and PB125 females NS no significant or trending difference

**Description**	**Male Control (n=14)**	**Male PB125 (n=14)**	**Female Control (n=14)**	**Female PB125 (n=14)**	**2-way ANOVA p-values**	**Bonferroni p-values**
Distance Traveled (m)	6.99; 5.11	9.54; 6.42	10.91; 7.47	7.46; 5.31	*0.0737[f]*	NS
Average Speed (m/s)	0.012; 0.009	0.016; 0.011	0.018; 0.013	0.013; 0.009	*0.0769[f]*	NS
Time Mobile (s)	89.58; 62.36	114.0; 78.03	115.3; 59.59	99.58; 72.88	NS	NS
% Time Mobile	14.93; 10.39	19.00; 13.01	19.22; 9.93	16.60; 12.15	NS	NS
Time in Hut (s)	282.2; 189.0	152.5; 170.9	260.6; 149.9	296.7; 186.3	0.0817[f]	*0.0670[Δ]* *0.1096[*]*
% Time in Hut	59.31; 31.94	25.41; 28.49	43.44; 24.99	37.18; 25.99	**0.0097[i]** *0.0703[f]*	**0.0046[*]**
