# Supplementary material for: Three-Month Administration of PB125 Modifies Histopathology, Redox Homeostasis, and Mobility in the Hartley Guinea Pig Model of Primary Osteoarthritis
Source: Antioxidants (Basel). 2026 Feb 5;15(2):212. doi: 10.3390/antiox15020212 (PMC12938315; doi:10.3390/antiox15020212)
Supplement: Supplementary file 1 [file antioxidants-15-00212-s001.zip › Supplemental Table S4 Digigait indices.pdf]

Supplemental Table S4. Digigait indices depicted as means and standard deviation from 5-month-old control and Nrf2-activator treated guinea pigs. Trending (*p<0.15, italic)* and significant (**p<0.05, bold**) sources of variation (determined by 2-way ANOVA, factors signifying sex and treatment) are listed. Trending *(p<0.15, italic)* and significant (**p<0.05, bold**) differences between groups (determined by Bonferroni multiple comparisons post hoc analysis) are listed in the last column.

2-way ANOVA key:

^{S}*sex*
$^{/}$*treatment*
^{f}*interaction*
NS no significant or trending source of variation identified

Bonferroni multiple comparisons key:

**Difference between control males and PB125 males*
$^{\infty}$*Difference between control females and PB125 females*
$^{\Psi}$*Difference between control males and control females*
$^{\Delta}$*Difference between PB125 males and PB125 females*
NS no significant or trending difference

Indice	Male Control (N=9)	Male PB125 (N=11)	Female Control (N=10)	Female PB125 (N=12)	2-way ANOVA P-values	Bonferroni P-values
**Swing**	0.100; 0.009	0.098; 0.009	0.092; 0.009	0.091; 0.010	**0.0158**^{8}	*0.1426*$^{\Psi}$
**%Swing Stride**	32.678; 2.849	31.580; 2.654	31.100; 2.747	31.938; 3.438	NS	NS
**Brake**	0.041; 0.015	0.037; 0.015	0.034; 0.018	0.045; 0.026	NS	NS
**%Brake Stride**	13.29; 4.44	11.98; 4.52	11.37; 6.23	15.46; 8.32	NS	NS
**Propel**	0.165; 0.013	0.175; 0.017	0.170; 0.019	0.150; 0.026	**0.0203**^{t} *0.1356*^{S}	**0.0403**$^{\infty}$ **0.0136**$^{\Delta}$
**%Propel Stride**	54.03; 4.53	56.07; 3.97	57.55; 5.32	52.60; 6.75	**0.0465**^{t}	*0.0682*$^{\infty}$
**Stance**	0.206; 0.015	0.212; 0.019	0.204; 0.017	0.195; 0.024	*0.1279*^{S}	*0.0988*$^{\Delta}$
**%Stance Stride**	67.32; 2.85	68.42; 2.65	68.90; 2.75	68.06; 3.44	NS	NS
**Stride**	0.306; 0.016	0.309; 0.021	0.296; 0.020	0.286; 0.028	**0.0199**^{8}	**0.0337**$^{\Delta}$
**%Brake Stance**	19.71; 6.30	17.36; 6.20	16.37; 8.64	22.37; 10.74	NS	NS
**%Propel Stance**	80.29; 6.30	82.64; 6.20	83.63; 8.64	77.63; 10.74	*0.1177*^{f}	NS
**Stance/Swing**	2.083; 0.263	2.190; 0.238	2.255; 0.285	2.175; 0.393	NS	NS
**Stride Length**	16.806; 0.866	17.015; 1.154	16.282; 1.106	15.692; 1.543	**0.0194**^{8}	**0.0308**$^{\Delta}$

**Stride Frequency**	3.317; 0.237	3.295; 0.243	3.445; 0.208	3.588; 0.405	**0.0250^{8}**	**0.0483$^{\Delta}$**
**Paw Angle**	-0.394; 1.748	-0.325; 3.315	-0.055; 2.518	-1.613; 3.029	NS	NS
**Absolute Paw Angle**	13.339; 2.906	11.745; 4.062	16.227; 5.314	14.821; 4.940	**0.0389^{8}**	NS
**Paw Angle Variability**	5.633; 4.679	6.415; 6.740	4.091; 1.945	7.138; 4.235	NS	NS
**Stance Width**	2.811; 0.549	3.000; 0.558	2.809; 0.464	2.900; 0.602	NS	NS
**Step Angle**	72.767; 5.151	68.990; 8.418	70.864; 4.351	72.400; 5.910	NS	NS
**Stride Length Variability**	2.612; 1.546	2.024; 1.506	1.418; 0.438	2.393; 1.258	*0.0500^{f}*	*0.0787$^{\Psi}$*<br>*0.1362$^{\infty}$*
**Stance Width Variability**	0.470; 0.305	0.319; 0.142	0.352; 0.164	0.448; 0.245	*0.0793^{f}*	NS
**Step Angle Variability**	7.177; 1.861	8.710; 7.628	6.595; 3.323	9.398; 3.111	*0.1285$^{/}$*	NS
**Number of Steps**	5.500; 0.820	6.100; 0.868	5.955; 0.748	5.458; 0.509	**0.0215^{t}**	*0.0977$^{\Delta}$*
**Stride Length CV**	15.443; 8.739	11.804; 8.496	8.744; 2.759	15.527; 8.414	**0.0303^{t}**	*0.1059$^{\Psi}$*<br>*0.0714$^{\infty}$*
**Stance Width CV**	16.790; 11.097	11.124; 5.838	13.233; 8.302	16.530; 10.723	*0.1265^{f}*	NS
**Step Angle CV**	9.959; 2.901	14.426; 17.257	9.386; 5.001	13.298; 5.255	NS	NS
**Swing Duration CV**	16.315; 9.942	16.890; 11.445	14.688; 8.212	13.829; 7.790	NS	NS
**Paw Area at Peak Stance (cm^2)**	3.717; 1.673	3.229; 0.700	2.717; 0.467	3.245; 1.130	*0.1319^{f}*<br>*0.1448^{S}*	*0.0865$^{\Psi}$*
**Paw Variability at Peak Stance**	0.413; 0.572	0.303; 0.296	0.198; 0.268	0.593; 0.670	*0.1039^{f}*	*0.1205$^{\infty}$*
**Hind Limb Shared Stance Time**	0.116; 0.018	0.136; 0.023	0.121; 0.026	0.114; 0.028	*0.0840^{f}*	*0.0846$^{\Delta}$*
**% Shared Stance**	56.39; 5.90	64.25; 11.71	58.99; 8.80	57.78; 7.86	*0.1069^{f}*	*0.1212**
**Stance Factor**	0.993; 0.037	0.972; 0.048	0.981; 0.039	0.966; 0.107	NS	NS
**Gait Symmetry**	1.050; 0.114	0.994; 0.044	1.014; 0.054	0.992; 0.028	*0.0586$^{/}$*	*0.1322**
**MAX dA/dT**	370.845; 155.355	325.387; 72.542	287.122; 40.530	310.653; 82.524	*0.0978^{S}*	*0.1068$^{\Psi}$*
**MIN dA/dT**	-71.012; 72.487	-51.365; 25.377	-47.649; 24.624	-58.261; 40.799	NS	NS

**Tau Propulsion**	0.140; 0.050	0.118; 0.023	0.124; 0.024	0.136; 0.036	*0.1156*^{f}	NS
**Overlap Distance**	-1.712; 2.204	-1.045; 1.089	-1.627; 1.437	-0.649; 1.569	*0.1058*$^{/}$	NS
**Ataxia Coefficient**	0.394; 0.246	0.311; 0.249	0.223; 0.082	0.404; 0.240	*0.0540*^{f}	*0.0999*$^{\infty}$
**Midline Distance**	4.116; 1.112	3.921; 0.816	3.830; 0.821	4.038; 0.794	NS	NS
**Axis Distance**	0.031; 0.221	0.137; 0.214	0.068; 0.250	0.200; 0.213	*0.0964*$^{/}$	NS
**Paw Drag**	-21.472; 4.876	-16.496; 4.609	-15.425; 4.708	-16.216; 14.003	NS	NS
