# Supplementary figures and images for: Three-Month Administration of PB125 Modifies Histopathology, Redox Homeostasis, and Mobility in the Hartley Guinea Pig Model of Primary Osteoarthritis
Source: Antioxidants (Basel). 2026 Feb 5;15(2):212. doi: 10.3390/antiox15020212 (PMC12938315; doi:10.3390/antiox15020212)

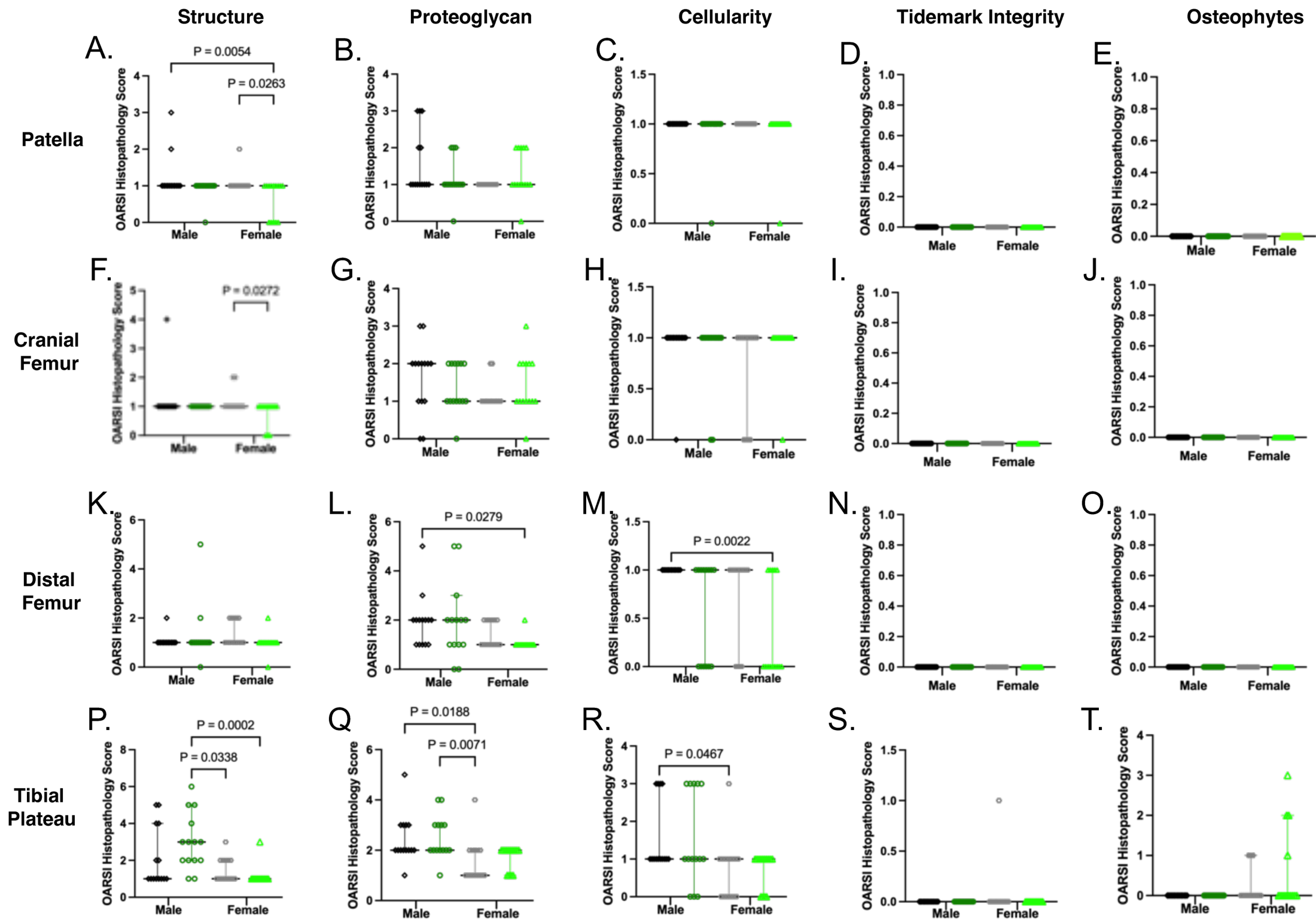

Supplement: Supplementary file 1 [file antioxidants-15-00212-s001.zip › SUPPLEMENTAL Figure S1 OARSI.pdf]

# Articular Cartilage

Nrf2

A.

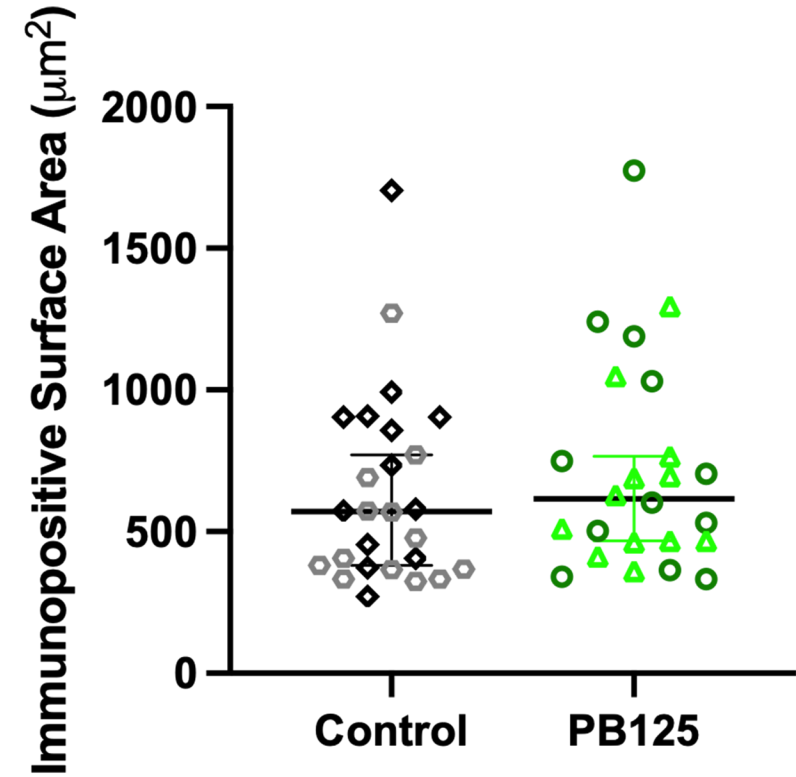

B.

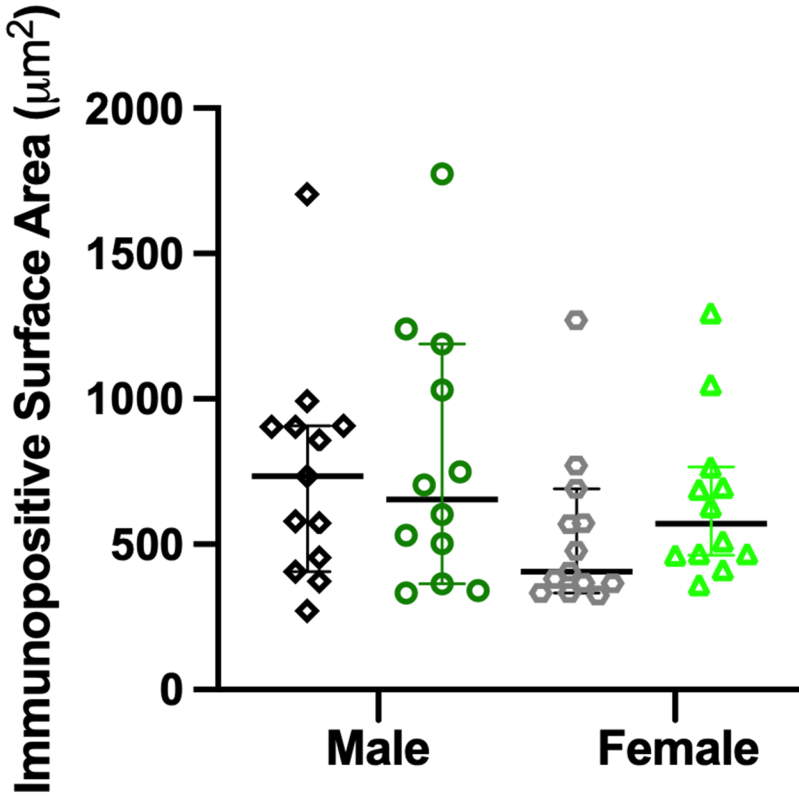

C.

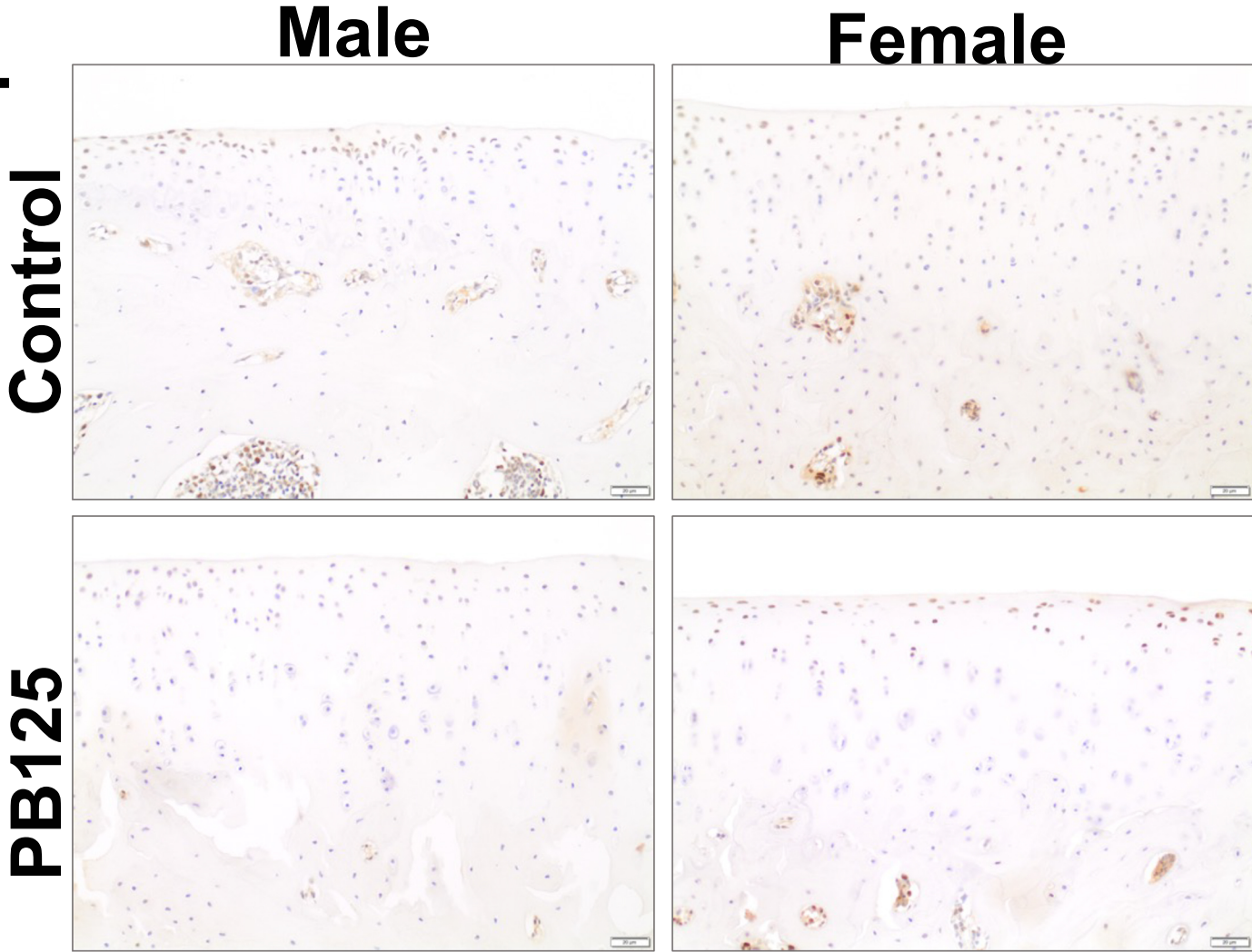

◇ Male Control    ○ Male PB125    ◇ Female Control    △ Female PB125

Supplement: Supplementary file 1 [file antioxidants-15-00212-s001.zip › Supplemental Figure S2 Nrf2.pdf]

# Articular Cartilage Patellar Surface

**A.**

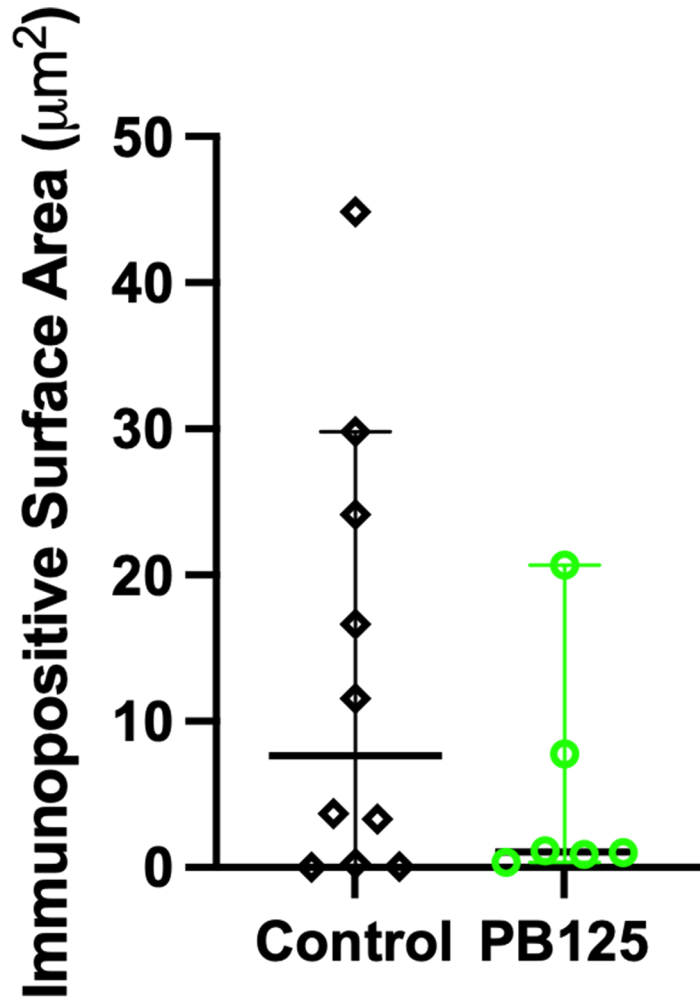

**B.**

# Male

# Control

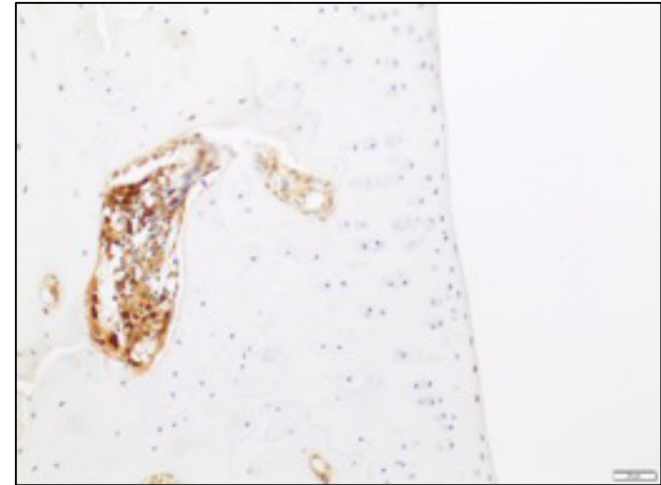

**PB125**

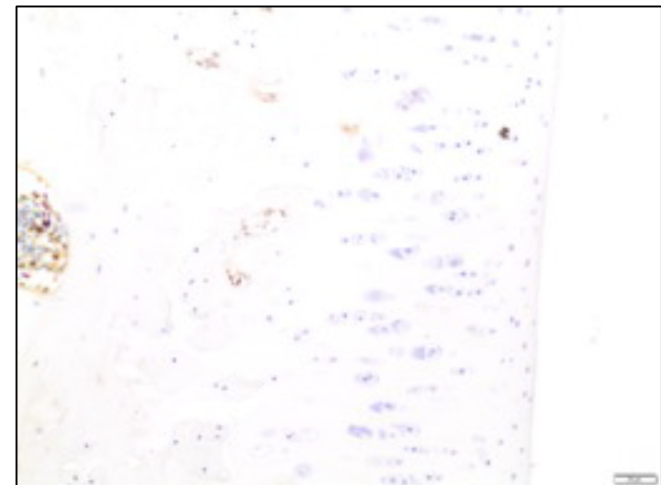

Supplement: Supplementary file 1 [file antioxidants-15-00212-s001.zip › Supplemental Figure S3 NQO1.pdf]
